# Microbial community dynamics over large spatial and environmental gradients in a subtropical ocean basin

**DOI:** 10.1128/aem.01889-25

**Published:** 2026-01-22

**Authors:** Sean R. Anderson, Katherine Silliman, Leticia Barbero, Fabian A. Gomez, Beth A. Stauffer, Astrid Schnetzer, Christopher R. Kelble, Luke R. Thompson

**Affiliations:** 1 Department of Biological Sciences, University of New Hampshire 3067, Durham, New Hampshire, USA; 2 Marine Chemistry and Geochemistry Department, Woods Hole Oceanographic Institution 10627, Falmouth, Massachusetts, USA; 3 National Oceanic and Atmospheric Administration, Atlantic Oceanographic and Meteorological Laboratory, Ocean Chemistry and Ecosystems Division, Miami, Florida, USA; 4 Northern Gulf Institute, Mississippi State University 621298 , Starkville, Mississippi, USA; 5 Cooperative Institute for Marine and Atmospheric Studies, University of Miami, Miami, Florida, USA; 6 National Oceanic and Atmospheric Administration, Atlantic Oceanographic and Meteorological Laboratory, Physical Oceanography Division 96721, Miami, Florida, USA; 7 School of Biological Sciences, University of Louisiana at Lafayette 4365, Lafayette, Louisiana, USA; 8 Marine, Earth, and Atmospheric Sciences, North Carolina State University, Raleigh, North Carolina, USA; University of Delaware, Lewes, Delaware, USA

**Keywords:** metabarcoding, generalized additive models, indicator analysis, microbes, protists, prokaryotes, environmental drivers, spatial dynamics

## Abstract

Microbes are fundamental to ocean ecosystem function, yet they remain understudied across broad spatial and environmental scales in dynamic regions like the Gulf of America/Gulf of Mexico (GOM). We employed DNA metabarcoding to characterize prokaryotes (16S V4–V5) and protists (18S V9) across 51 stations, spanning 16 inshore–offshore transects and three depths. Cluster analysis revealed three clusters corresponding to depth zones that integrated vertical and horizontal sampling: photic zone (inshore near surface–bottom and offshore surface), deep chlorophyll maximum (offshore), and aphotic zone (offshore near bottom). We applied group-specific generalized additive models (GAMs) to log-transformed abundance data of major taxa in the photic zone, identifying key environmental factors that explained 42%–82% of the variation in abundance. SAR11 and SAR86 were positively associated with temperature and dissolved inorganic carbon, while cyanobacterial genera (*Prochlorococcus* and *Synechococcus*) were differently impacted by nutrients, salinity, and pH in ways that often followed their expected ecological niches. Representatives of protist parasites (Syndiniales) and grazers (Sagenista) showed group-specific nonlinear associations with salinity, oxygen, nutrients, and temperature. Using GAMs, we expanded the spatial resolution of DNA sampling and predicted surface log abundances at 84 cruise sites lacking amplicon data. Indicator analysis was performed with sequence-level data, revealing several protists that were indicative of more acidic waters and the absence of any significant prokaryote indicators. Our results provide the first basin-scale survey of microbes in the GOM and highlight the need for coordinated omics and environmental sampling to improve predictions of microbial responses to changing conditions.

**IMPORTANCE:**

Marine microbes are key indicators of environmental change and play central roles in ocean food webs and biogeochemical cycles. Yet, how natural microbial communities respond to shifting environmental conditions remains unclear, particularly in the Gulf of Mexico (GOM), a region shaped by dynamic physical and chemical gradients. Here, we conducted a novel basin-scale DNA metabarcoding survey of prokaryotes and protists in the GOM. We used generalized additive models and indicator analysis to reveal environmental drivers of microbial abundance, from broader taxonomic groups to unique sequences. Our results show group-specific associations with environmental factors such as temperature, nutrients, salinity, and carbonate chemistry parameters and identify several protist taxa associated with distinct ocean conditions. These findings provide a foundation for microbial monitoring in the GOM and shed light on the importance of integrating *in situ* biological, physical, and chemical data across spatial gradients to inform accurate ecosystem and biogeochemical models.

## INTRODUCTION

Microbes form the base of ocean food webs, driving primary production, mediating global biogeochemical cycles, and supporting marine fisheries and other essential ecosystem services (1, 2). In addition to biological interactions, the composition and activities of microbial communities are influenced by a range of environmental drivers, including temperature, salinity, nutrient availability, and oxygen levels, all of which vary over time and space (3, 4). Though not as well understood, ocean acidification (OA), the process whereby increasing atmospheric CO_2_ (~420 ppm at present) leads to increased levels of dissolved inorganic carbon (DIC) in the ocean, can also alter microbial physiology, community composition, and biodiversity (5–7). Furthermore, the impacts of OA on microbes can potentially have cascading effects on marine food webs and carbon cycling (8). Ultimately, there is a need to better understand how different abiotic factors shape natural microbial communities to predict how ecosystems will respond to environmental changes.

In general, global ecosystem models predict a decline in photosynthetic biomass and a shift in composition from large plankton (e.g., diatoms) to picophytoplankton (0.2 µm–2 µm), primarily driven by warming and enhanced stratification (9–12). In terms of OA, field and laboratory experiments have measured direct and negative impacts of increased partial pressure of CO_2_ (*p*CO_2_) on certain phytoplankton groups, most notably reduced growth and calcification rates among calcifying haptophytes (13–15). However, resiliency to OA has also been observed, even at the species to strain level (16, 17). Compared to protists, bacteria may be more resilient to OA and more directly impacted by increased temperature and changes in phytoplankton-derived organic matter (18, 19). Despite these findings, the outlook for diverse microbial groups remains unresolved, especially for protists, many of which are uncultured and therefore are missed in sample analyses that rely on morphology. There is a need to employ more highly resolved methods, such as DNA metabarcoding (20, 21), to effectively capture *in situ* microbial dynamics, which will help to guide future lab- and field-based studies, inform model predictions, and identify microbial signatures of different ocean conditions.

The Gulf of America/Gulf of Mexico (GOM) is a dynamic environment in which to study microbes and their environmental drivers (22). As a semi-enclosed subtropical basin, the GOM is shaped by many hydrographic features, including the Loop Current (and associated anticyclonic eddies), freshwater inflow from major riverine systems (Mississippi–Atchafalaya), and coastal upwelling that delivers nutrients and carbon onto the shelf (23, 24). These features can elicit strong gradients in temperature, salinity, nutrients, and oxygen levels, in turn influencing the structure and function of microbial communities across spatial and temporal scales (25). Though most of the GOM is oligotrophic (and nutrient-limited), episodic nutrient loading from terrestrial sources can promote coastal eutrophication that leads to hypoxic zones that are more acidic (26, 27). Eutrophication combined with wind-driven upwelling of nutrients on the shelf can also enhance primary production and at times lead to the formation of harmful algal blooms, particularly along Florida’s western coast (28) and other coastal sites in the southern GOM (29). Lastly, there is strong evidence of OA in surface waters of the open GOM (e.g., increased *p*CO_2_) that is on par with rates of change measured at oligotrophic sites in the Atlantic and Pacific Oceans (30). Together, dynamic physical and chemical conditions in the GOM create an ideal system to explore microbial biogeography with respect to natural and anthropogenic pressures.

Though environmentally variable, microbial communities in the GOM have not been well characterized at the basin scale (22). Most microbial omics studies in the GOM have been localized to specific regions or depths (31–35) or focused on responses of microbes to natural disturbances (e.g., oil spills) in the northern GOM (36, 37). Though much of the open-ocean GOM has low biomass, microbial food webs are known to support high biodiversity (and biomass) of larger organisms (zooplankton to fish) throughout the water column (38–40), making it important to better resolve microbial distributions over spatial scales. A lack of spatially inclusive biological sampling has made it difficult to characterize environmental drivers of microbes in the GOM and impedes our ability to understand how microbial communities, and the ecosystem processes they underpin, may shift in the future.

**Fig 1:**
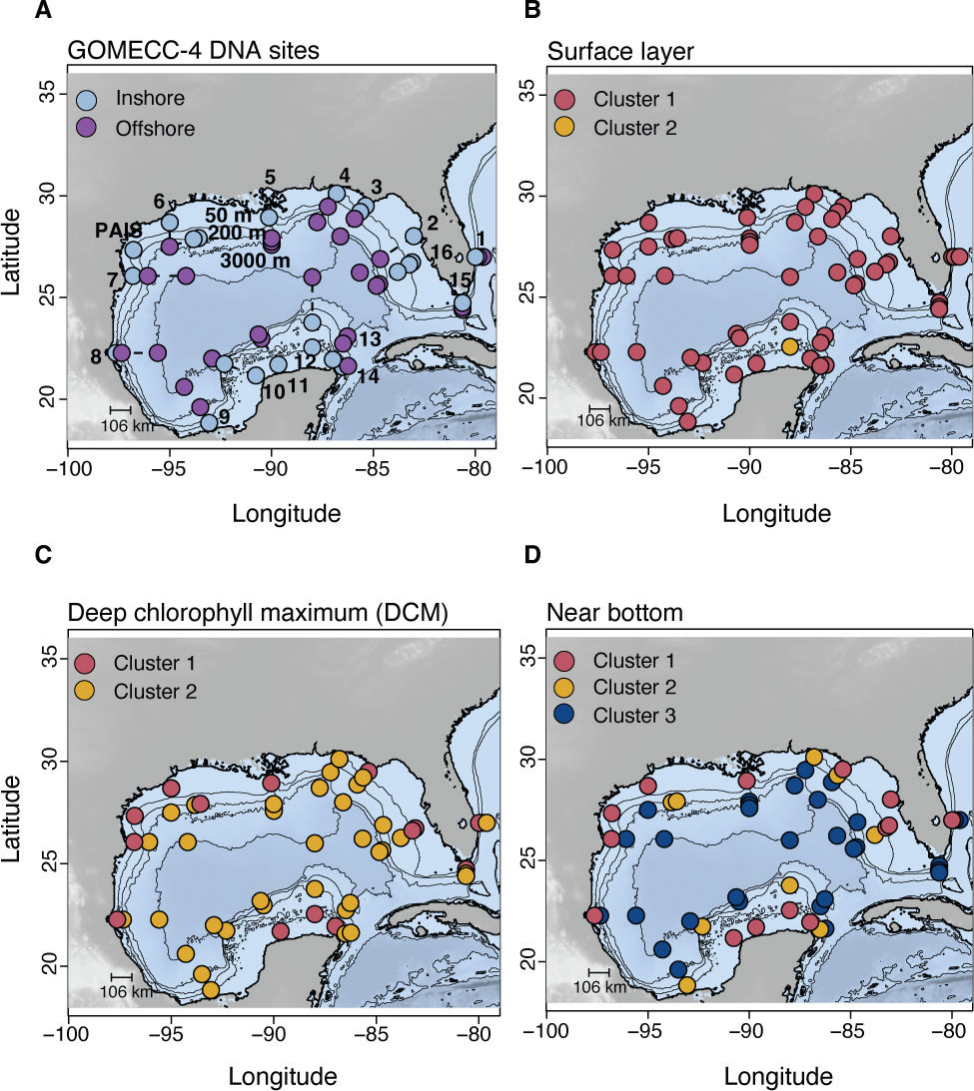
Basin-scale DNA sampling in the GOM. (**A**) DNA samples were collected on 16 transects that included sites inshore on the continental shelf (light blue; <200 m) and in offshore waters >200 m (purple). The numbers identify the different transects (or lines) that were sampled in chronological order: (1) 27°N, (2) Tampa, (3) Panama City, (4) Pensacola, (5) Louisiana, (6) Galveston, (7) Brownsville, (8) Tampico, (9) Veracruz, (10) Campeche, (11) Merida, (12) Yucatan, (13) Catoche, (14) Cancun, (15) Florida Straits, and (16) Cape Coral. Three depths were sampled per site, which represented the surface layer, DCM, and near bottom. Samples were collected in triplicate (shown as a single point), and environmental data were collected from all sites. An additional site was included at Padre Island National Seashore (PAIS). (**B–D**) The same samples in panel A, now colored by their respective 18S cluster assignment, show lateral distribution patterns at the surface layer (**B**), DCM (**C**), and near bottom (**D**). Hierarchical clustering of 16S samples followed a similar pattern, and cluster maps for 16S are shown in Fig. S7.

In this study, we performed basin-scale DNA metabarcoding in the GOM as part of the fourth Gulf of Mexico and East Coast Carbon (GOMECC-4) cruise that sailed from late summer to early fall in 2021. Overall, we collected 481 discrete DNA samples from 51 (out of 141) stations, encompassing 16 inshore–offshore transects and up to three depths per site that corresponded to the surface, deep chlorophyll maximum (DCM), and near bottom (Fig. 1). Amplicon metabarcoding was performed to reveal the population dynamics of protists (18S SSU rRNA gene, V9 region) and prokaryotes (16S SSU rRNA gene, V4–V5 region) at previously unresolved spatial scales in this region. Using log-transformed abundances, we constructed generalized additive models (GAMs) for each of the major microbial groups in the photic zone to gain insight into group-specific environmental predictors, which included temperature, salinity, oxygen, dissolved nutrients, pH, and DIC. Models were applied to all cruise sites, including 84 sites where DNA samples were not collected, to predict microbial log abundances and expand the spatial DNA sampling resolution in the GOM. Finally, we performed indicator analysis based on profiles of total alkalinity (TA) and DIC in the photic zone, which revealed microbial signatures of different acidic conditions (based on TA:DIC ratios). Our findings expand the spatial scope of microbial dynamics in the GOM and underscore the need for sustained biogeochemical monitoring in this region, with implications for accurate modeling, resource management, and ecosystem health.

## MATERIALS AND METHODS

### Sample collection

Seawater was collected on board the NOAA Ship *Ronald H. Brown* as part of GOMECC-4 from 13 September to 21 October 2021. Sampling for GOMECC-4 occurred along 16 inshore–offshore transects across the entire GOM and an additional line at 27°N latitude in the Atlantic Ocean (Fig. 1A). Sampling began at the 27°N line and continued counterclockwise, ending at sites in the Florida Straits and off the coast of Cape Coral, Florida. We also collected DNA samples near Padre Island National Seashore (US National Park Service), a barrier island located off the coast of south Texas (Fig. 1A). Vertical CTD sampling was employed at each site to measure discrete physical, chemical, and biological properties. Water sampling for DNA filtration was conducted at 51 out of 141 total sites and at three depths per site, the latter varying based on total water depth (i.e., position on shelf or offshore) but always representing a surface (2 m–10 m), DCM (9 m–99 m), and near-bottom sample (9 m–3,326 m).

At each respective site and depth, seawater was collected from pre-designated Niskin bottles on a CTD rosette. To ensure adequate amounts of water were filtered for DNA analysis, samples for chemical parameters were taken at the same depths but with different discrete Niskin bottles. Following a CTD cast, which varied in duration from 30 min to 3 h depending on water depth, whole seawater was transferred from Niskin bottles to triplicate Whirl-Pak bags (3 depths × 3 replicates = 9 bags per site). Within an hour, seawater (~2 L per replicate) was filtered through 0.22-µm Sterivex filters (Millipore; CAT# SVGP01050) via a peristaltic pump (100 rpm–150 rpm) and run dry. Filters were capped, sealed with parafilm, and stored at −80°C on the ship and at the same temperature for longer-term storage in the lab. Filter lines were sterilized with 2% bleach and rinsed with Milli-Q after every site. Milli-Q blanks were also filtered randomly throughout the cruise. Accounting for blanks and replication, 481 total Sterivex filters were collected.

Discrete samples for water column hydrography and chemistry were collected alongside DNA samples. Temperature, salinity, pressure, and chlorophyll fluorescence were obtained from the CTD. Vertical CTD profiles on the downcast were used to estimate the position of the DCM at each site. Water samples were collected for dissolved oxygen, nutrients (nitrate = NO_3_, nitrite = NO_2_, ammonium = NH_4_, phosphate = PO_4_, and silicate = SiO_4_), DIC, TA, *p*CO_2_, pH, and carbonate ion concentration. Additional details on sample metadata collection and processing can be found in the cruise report (41) and in the Supplemental Material file. Aragonite saturation state was calculated at each site and depth based on temperature, salinity, pressure, DIC, and TA using the CO2SYS program for CO_2_ system calculations (42). Measurements of *p*CO_2_ (20°C) and pH (25°C) were re-calculated to *in situ* conditions using pressure, temperature, salinity, DIC, and TA in CO2SYS. Metadata associated with DNA samples are provided in Table S1.

### DNA extractions, PCRs, and library preparation

Sterivex filters were extracted at NOAA’s Atlantic Oceanographic and Meteorological Laboratory (AOML) using the ZymoBIOMICS 96 MagBead DNA Kit (Zymo; CAT# D4308), with modifications for in-cartridge bead beating (43). Filters were thawed, the inlet caps removed, and excess water was dried from the inlet using Kimwipes to dispense beads into the cartridge. Premade mixtures of 0.1-mm and 0.5-mm beads were directly added into the filters to ensure adequate lysis and recovery of hard-to-lyse phytoplankton groups (43). This was followed by the addition of a lysis buffer mixture from the kit (1 mL). Filters were vortexed for 40 min on a Vortex-Genie at maximum speed (~3,200 rpm). DNA lysates were transferred to 2-mL LoBind tubes (Eppendorf) via syringe and centrifuged for 1 min at 10,000 *g*. Supernatant (750 µL per sample) was split across three KingFisher 96-well plates (250 µL per plate). Zymo MagBinding buffer (600 µL) and magnetic beads (25 µL) were added to each well in each of the three plates. With this setup, 96 samples were extracted at the same time on the automated KingFisher Flex (Thermo Fisher). Each run included three wash plates with 500 µL–900 µL per well of MagWash and an elution plate with 150 µL per well of molecular-grade water. DNA was eluted into a single well from the same discrete sample across replicate plates. Concentrations of eluted DNA were measured using a Varioskan LUX plate reader and the Quant-IT dsDNA Assay (Thermo Fisher) and corrected per replicate sample based on volume of seawater filtered (nanograms per liter; Table S1). Filters were processed randomly, and replicates were not pooled. Extraction blanks (clean filters) were also included and processed similarly. The ZymoBIOMICS Microbial Community DNA Standard (Zymo; CAT# D6306) was included as a positive control.

Metabarcoding libraries were initially prepared at AOML, amplifying DNA of target organisms with universal primers, including 16S (Bacteria and Archaea) and 18S rRNA (protists). Primers from (44) were used to target the 16S V4–V5 region: forward (515F-Y; 5′-GTGYCAGCMGCCGCGGTAA-3′) and reverse (926R; 5′-CCGYCAATTYMTTTRAGTTT-3′). The predicted length of 16S amplicons after removing primers was ~373 bp. Primers from (45, 46) targeted the 18S V9 region: forward (1391F; 5′-GTACACACCGCCCGTC-3′) and reverse (EukB; 5′-TGATCCTTCTGCAGGTTCACCTAC-3′). The predicted length of 18S amplicons after removing primers was ~126 bp. Other 18S primers, such as those targeting the V4 region, cover a larger section of the 18S rRNA gene (~378 bp after removing primers) and may offer greater taxonomic resolution and recovery of rare eukaryotic taxa (47). However, others have found both primer sets to perform similarly well for 18S samples (48), and in our case, we used V9 primers to align with global marine surveys (e.g., Tara Oceans and Earth Microbiome Project). Both primer sets were constructed with Fluidigm common oligos CS1 forward (CS1-TS-F: 5′-ACACTGACGACATGGTTCTACA-3′) and CS2 reverse (CS2-TS-R: 5′-TACGGTAGCAGAGACTTGGTCT-3′) fused to their 5′ ends, to enable dual indexes to be added in a second PCR step at the Michigan State University Research Technology Support Facility (RTSF).

PCR reactions were run in triplicate (13 µL per sample), with 1 µL of DNA per sample. 16S PCR reactions consisted of 5 µL of AmpliTaq Gold, 6.25 µL of water, and 0.375 µL of each primer (10 µM); PCR conditions included denaturation at 95°C for 2 min, 25 cycles of 95°C for 45 s, 50°C for 45 s, and 68°C for 90 s, followed by a final elongation step of 68°C for 5 min (44). 18S PCR reactions consisted of 5 µL of AmpliTaq Gold, 6.5 µL of water, and 0.25 µL of each primer (10 µM); PCR reactions involved denaturation at 94°C for 3 min, 35 cycles of 94°C for 45 s, 65°C for 15 s, 57°C for 30 s, and 72°C for 90 s, followed by a final elongation step of 72°C for 10 min (46). PCR products were pooled and run on a 2% agarose gel to confirm amplification of target bands. Sample plates were submitted to the Michigan State University RTSF Genomics Core for secondary PCR and sequencing.

Secondary PCR used dual-indexed, Illumina-compatible primers, targeting the Fluidigm CS1/CS2 oligomers at the ends of the PCR products. PCR conditions for the secondary run included an initial denaturation step at 95°C for 3 min, 11 cycles of 95°C for 15 s, 60°C for 30 s, and 72°C for 60 s, followed by elongation at 72°C for 3 min. Amplicons were batch-normalized using Invitrogen SequalPrep DNA Normalization plates, and the recovered product was pooled. The pool was QC’d and quantified using a combination of Qubit dsDNA HS, Agilent 4200 TapeStation HS DNA1000, and Invitrogen Collibri Library Quantification qPCR assays. The RTSF Core included a sequencing blank for each sample plate. Separate sequencing runs were performed using an Illumina MiSeq (2 × 250 bp) for 18S and 16S samples. Custom sequencing and index primers complementary to the Fluidigm CS1 and CS2 oligomers were added to appropriate wells of the reagent cartridge. Base calling was done by Illumina Real Time Analysis (RTA) v.1.18.54, and output of RTA was demultiplexed and converted to FASTQ format with Illumina Bcl2fastq v.2.20.0.

### Bioinformatics

Primers were removed from demultiplexed FASTQ sequences using Cutadapt (49). Trimmed reads were processed in Tourmaline, which implements QIIME 2 (and the DADA2 plugin) in a Snakemake workflow (50). Paired-end DADA2 was used to infer 16S and 18S amplicon sequence variants or ASVs (51). Taxonomic assignments were also performed in Tourmaline using reference files from SILVA (version 138.1 [52]) and the Protistan Ribosomal Reference or PR2 (version 5.0.1 [53]) databases for 16S and 18S ASVs, respectively. In both cases, taxonomy was assigned using a Naïve Bayes classifier trained on the respective databases and trimmed to the primer regions (54). Output files (taxonomy, count, and metadata) were imported separately into R (version 4.4.2) using qiime2R (version 0.99.6; https://github.com/jbisanz/qiime2R) and merged with phyloseq (version 1.50.0 [55]).

Several non-protistan eukaryotic groups were removed from the 18S data set, including Metazoa, Streptophyta, Rhodophyta, Insecta, Archosauria, and Ascomycota. Unassigned reads at the subdivision level were also filtered out. For 16S, reads assigned to Chloroplast, Mitochondria, and Eukaryota were removed. Samples with fewer than 3,000 read counts were filtered out for 18S (5,000 reads for 16S), along with ASVs only observed once in each respective data set. After these initial filtering steps, 461 and 465 out of 481 samples (inclusive of blank removal) remained for 18S and 16S data sets, respectively. Species accumulation curves were generated using the R package ranacapa (version 0.1.0 [56]). The number of reads vs ASVs was saturated for most samples, which indicated an appropriate sequencing depth was reached (Fig. S1). Samples were rarefied to the minimum read count for protists (3,318) and prokaryotes (5,056) to normalize for differences in library size. Protist ASVs were manually assigned to functional groups based on 18S V9 functional annotations that were previously applied to Tara Ocean communities (57). Additional databases (e.g., World Register of Marine Species) and literature searches were used. The following functional groups were included for protists: autotrophic, heterotrophic, mixotrophic, parasites, photosymbionts, and other protists. Additional details on functional assignments are in the Supplemental Material file. All functional assignments are in Table S2.

### Sample clustering and statistical analyses

To better observe spatial patterns without prior assumptions, samples were grouped into clusters via hierarchical clustering (Ward’s method) using the hclust function in vegan (version 2.6-10 [58]). Prior to clustering and ordination, ASV count tables were transformed to Aitchison distances, which are estimated by transforming read counts via centered log-ratio (CLR) normalization and computing Euclidean distances. The optimal number of clusters was determined based on average silhouette widths using the factoextra package (version 1.0.7 [59]). Silhouette widths offer an estimate of the quality of sample clustering, with higher width coefficients indicating optimal clustering (60). Microbial composition was observed via principal coordinates analysis (PCoA) of Aitchison distances. In addition, the influence of categorical factors on composition, such as depth (surface, DCM, and near bottom), distance to shore (inshore vs offshore), and sampling transect, was tested for significance with permutational multivariate analysis of variance (PERMANOVA) using the adonis2 function in vegan with 999 permutations (58).

Shannon diversity index and richness (# of ASVs) were determined for each cluster using the estimate_richness function in phyloseq (55), and mean values were compared against each other to test for significance with Wilcoxon tests (*P* < 0.05; Holm-adjusted). Diversity and richness were also estimated along transects and with absolute depth, applying local regression (loess) curves to visualize spatial trends in the data sets with the geom_smooth function in ggplot2 (version 3.5.1 [61]). Stacked bar plots displaying mean relative abundance were observed in ggplot2 at the class level for 18S and order level for 16S and faceted to show general trends over spatial scales along each transect. Taxonomic profiles were also observed using the treemap package in R (version 2.4-4 [62]), a tiered approach to visualize relative abundance across multiple taxonomic levels.

Indicator taxa that were more abundant and representative of high (or low) TA:DIC ratios were statistically inferred using the indicspecies package in R (version 1.7.15 [63]). Briefly, indicator analysis aims to assess the strength and statistical significance of the relationship between species occurrence and groups in the data set (63). It tests whether taxa occur more frequently in particular sample groups than would be expected by chance and uses permutations (in this case 999) to test for significance (63). The TA:DIC ratio was chosen because it is a good proxy to determine the ocean’s capacity to absorb anthropogenic CO_2_ by influencing its buffering capacity (64). Higher ratios indicate strong buffering capacity (i.e., the capacity of seawater to buffer against acidification). Based on histograms of TA:DIC, samples were grouped into high (>1.16) or low categories (<1.16) that reflected different OA conditions. The cutoff of 1.16 was chosen after manual inspection of histogram density, suggesting a natural division in the data. Indicator tests were run separately on rarefied 16S or 18S samples that were agglomerated to the ASV level. Indicator values were filtered for significance (*P* < 0.05) and plotted against their mean ASV relative abundance in the photic zone. Different multiple-comparison corrections were applied to different analyses to adjust *P*-values. Holm correction was used for diversity metrics and model-based tests to conservatively limit false positives, whereas Benjamini–Hochberg correction was applied to indicator analyses to allow exploration of significant taxa across a larger ASV data set while controlling the false discovery rate.

### GAMs

To investigate the relationship between environmental factors (predictors) and group-specific microbial abundances (response) in the GOM, we employed generalized additive models (GAMs) with a Gaussian distribution to model log-transformed microbial abundance data. GAMs are well suited for ecological data because they allow for nonlinear, non-parametric relationships between the response and predictor variables without needing to specify the functional form of the data beforehand (65). Compared to generalized linear models (GLMs), which assume linearity, GAMs offer improved flexibility and performance when species-environment relationships are expected to exhibit nonlinear patterns, which is often the case among marine microbes (66). GAMs have been used previously in the GOM to predict species-specific fisheries counts (67, 68) and have also been applied to predict phytoplankton biomass (69) and bacterial abundances (70) in other ocean systems.

Separate GAMs were performed for the top 4 most relatively abundant order-level 16S and class-level 18S groups across all photic zone samples. Models were constructed for *Synechococcus* and *Prochlorococcus* separately to resolve differences between major cyanobacteria genera. Prior to running Gaussian GAMs, we aggregated ASV read counts for major microbial groups to their relevant taxonomic rank (class level for protists; order and genus for prokaryotes) using the tax_glom function in phyloseq (55), which sums counts across all ASVs belonging to that rank within each sample. These group-level abundances were merged with metadata, preserving sample replication. We log-transformed the summed and rarefied abundance values (with pseudo-count +1) to reduce variance heterogeneity and improve model fit (65). Models focused on photic zone samples to minimize collinearity among environmental predictors, which became more pronounced with depth (Fig. S2 and Table S3), and to take advantage of the larger number of surface samples available for reliable model training and validation. Gaussian GAMs performed better, as indicated by lower Akaike information criterion (AIC) values for all groups tested, than GAMs using raw counts with a negative binomial distribution or GLMs with negative binomial or Poisson distributions (Table S4). Residuals from the final Gaussian GAMs were approximately normally distributed, and Q–Q plots showed strong adherence to theoretical quantiles across all microbial groups. These diagnostics, visualized in Fig. S3, confirmed that model assumptions were met and supported the use of Gaussian models with log-transformed amplicon data.

To assess whether compositional bias influenced GAM results, we applied a CLR transformation (with a pseudo-count +1) to the abundance data and reran GAMs for all 16S and 18S groups using the same non-collinear environmental predictors. We compared CLR and log-transformed models using AIC, percent deviance explained, adjusted *R*^2^, and 1:1 predicted vs observed fits. Log-transformed models consistently had lower AIC values across microbial groups (Table S4). Except for SAR11 and *Synechococcus*, CLR-based GAMs resulted in weaker model performance than log-transformed models, with lower deviance explained and weaker 1:1 fits for the remaining microbial groups (Fig. S4 and S5), driven by increased clustering of samples near zero. Log abundance was therefore retained for model analyses.

Several environmental predictors, such as temperature, salinity, PO_4_, NH_4_, and NO_3_, were log-transformed (with pseudo-count +0.1) to reduce skewness, in part driven by sites near the Mississippi River outflow (Louisiana line). These coastal sites reflected real ecological data, and so we chose to retain them and transform these variables (over their removal) to capture basin-scale dynamics. However, we removed extreme outliers from samples collected on the 27°N transect, a region that extends beyond the GOM and can have distinct chemical properties. Outliers were identified using a strict interquartile range (IQR) method applied across all predictor variables. This resulted in the removal of two samples from the 18S data set and six from the 16S data set, leaving 228 and 261 photic zone samples, respectively (including replicates). Other predictors that were more normally distributed (oxygen, pH-corrected, and DIC) remained untransformed. Prior to modeling, we screened predictors for collinearity using Spearman’s rank correlation and variance inflation factor (VIF) analysis, retaining only variables with Spearman *r_s_* < |0.7| and VIF < 10 (71). Even after outlier removal and normalization, some predictors had sparse data points at the extremes that led to higher uncertainty (or curvature) in GAM partial effects, and these patterns should therefore be interpreted more cautiously.

We employed a stratified approach for model testing, splitting the 18S and 16S data into 80% training and 20% test sets for each major taxonomic group that was sensitive to region and log abundance. This ensured spatial heterogeneity and that a range of log-abundance values were represented in both subsets. For each microbial group, we ran 10-fold cross-validation on the training set, evaluating all non-empty combinations of predictor variables. Cross-validation performance was assessed using root mean square error (RMSE), and the best GAMs were implemented in the mgcv package in R (version 1.9-3 [72]). The restricted maximum likelihood method was used to select smoothing parameters (with default maximum complexity of *k* = 10). GAMs also used the select = TRUE option, which applies an extra penalty that can shrink overly flexible smooths towards a linear function or remove unsupported terms entirely. Together, these features helped to control model complexity, penalize uninformative terms, and ensure stable and interpretable smoothness estimation (65). The resulting final GAMs were used to predict log abundance in the test set, with model performance evaluated via RMSE. We also estimated percent deviance explained and adjusted *R*² for the training set as measures of model fit. Lastly, to visualize model performance, we plotted observed vs predicted log-abundance values for both training and test sets, overlaid with a 1:1 line to assess the alignment of predictions with actual values.

After fitting the final GAMs, we retained the full set of smooth terms identified through cross-validation when generating predictive maps at unsampled sites, regardless of individual term significance. In most cases, all smooth terms were significant, and at most one or two terms per microbial group were non-significant. These terms were nonetheless retained because they contributed to overall predictive performance, and removing them would alter the fitted relationships. These full models were used to estimate log abundance at all GOMECC-4 sites where surface layer (<10 m) variables were collected (135 out of 141 sites), which included 84 sites where DNA was not sampled. Six stations did not have representative CTD data available at the surface and were excluded. Predictors in this new data set were log-transformed as needed to match training conditions. Predicted log abundances were visualized using Data-Interpolating Variational Analysis (DIVA) in Ocean Data View (73) to map trends across the basin. Other GOM maps were constructed with the marmap package in R (version 1.0.10 [74]). To interpret variable effects within each model, we evaluated the statistical significance of smooth terms (*P* < 0.05; Holm-adjusted) using model summary outputs and generated partial effect plots with the gratia package in R (version 0.10.0 [75]). These plots emphasized significant predictors and were ordered based on effective degrees of freedom (EDF), indicating linear and nonlinear relationships (EDF ~1 indicates strong linearity). Model assumptions were assessed using gam.check(), which included diagnostic plots to evaluate residual distributions (Fig. S3).

## RESULTS AND DISCUSSION

### Microbial population dynamics in the GOM

Basin-scale DNA metabarcoding in the GOM revealed extensive microbial diversity, yielding a total of 41,876 16S and 13,632 18S ASVs, respectively. Sampling occurred along 16 inshore–offshore transects and three depths per station, spanning the surface layer, DCM, and near bottom (Fig. 1). Although factors such as transect, distance from shore (shelf vs offshore), and categorical depth were significantly associated with microbial composition (*P* < 0.01; Holm-adjusted), each factor had low explanatory power on its own (PERMANOVA *R*^2^ = 0.03–0.2). Therefore, to better capture the integrated effects of vertical and horizontal gradients, we applied hierarchical clustering to the community composition data. Through this approach, we identified ecologically relevant sample groupings without imposing any prior assumptions on the data structure.

**Fig 2:**
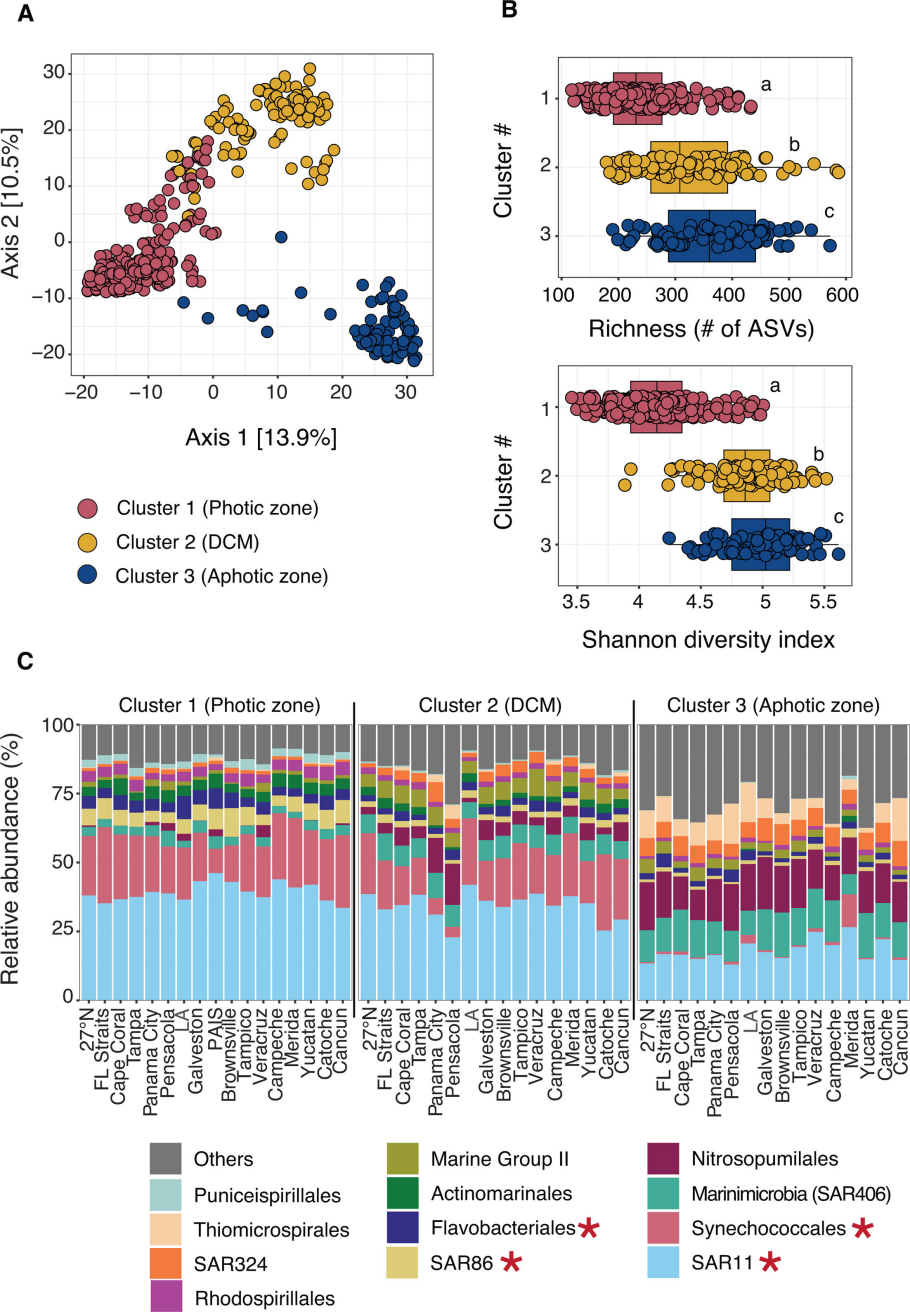
Bacterial and archaeal community dynamics in the GOM from 16S metabarcoding. (**A**) PCoA of Aitchison distances, with samples colored by their respective clusters. Clusters were determined via hierarchical clustering: Cluster 1 (photic zone), Cluster 2 (DCM), and Cluster 3 (aphotic zone). (**B**) Boxplots displaying observed richness (# of ASVs) and Shannon diversity index for Clusters 1–3, with points representing individual samples. Boxplots show median values (horizontal line), with boundaries representing the interquartile ranges (25%–75%) and whiskers extending to 1.5× the IQR. Data falling outside the 1.5× IQR are considered outliers in the plot. Significant differences in mean values between clusters are indicated by letters and were determined with Wilcoxon tests and corrected for multiple comparisons (Holm method; *P* < 0.05). (**C**) Stacked bar plots of mean relative abundance (%) at the order level in each sampling transect and faceted by cluster. Transects are ordered on the *x*-axis by the order of sampling (counterclockwise) on GOMECC-4, except for FL Straits and Cape Coral, which were sampled last but grouped spatially with other FL lines. Bar plots display the top 12 most relatively abundant groups over all samples (“others” in gray). Taxonomy was assigned via the SILVA database. GAMs focused on the top 4 most relatively abundant groups in Cluster 1 (red asterisks). Models for Synechococcales were constructed at the genus level to discriminate between *Prochlorococcus* and *Synechococcus*. LA, Louisiana; PAIS, Padre Island National Seashore. Transects have the same labels in all subsequent plots.

**Fig 3:**
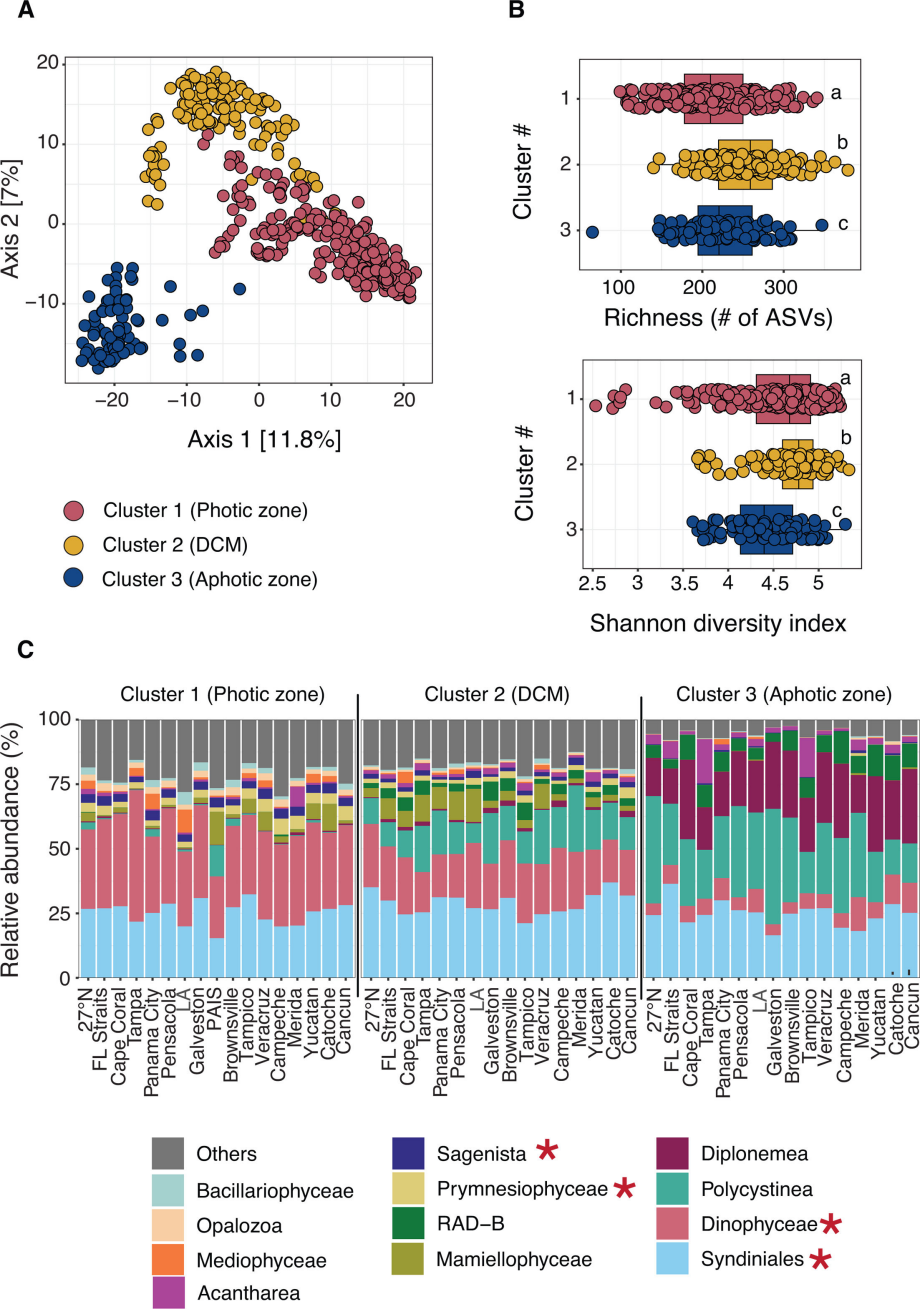
Protist community dynamics in the GOM. (**A**) PCoA plot of Aitchison distances, with 18S samples colored by cluster. (**B**) Boxplots as in Fig. 2 showing observed richness (# of ASVs) and Shannon diversity index for Clusters 1–3, with points representing individual samples. Significant differences in mean values between clusters (letters) were determined with Wilcoxon tests and corrected for multiple comparisons (Holm method; *P* < 0.05). Other details are the same as in Fig. 2. (**C**) Stacked bar plots of mean relative abundance (%) at the class level in each sampling transect and faceted by cluster. Transects are ordered the same as in Fig. 2. Bar plots display the top 12 most relatively abundant groups over all samples (“others” in gray). Protist taxonomy was assigned via the PR2 database. GAMs focused on the top 4 most relatively abundant groups in Cluster 1 (red asterisks).

Clustering analysis revealed three clusters that were consistent across both 18S and 16S data sets (Fig. S6), with 93% overlap in samples that implied similar patterns between prokaryote and protist communities (Fig. 1B through D; Fig. S7). Cluster 1 (*n* = 270 for 16S; 233 for 18S) included samples in the photic zone (2 m–99 m) from both shelf and offshore surface waters (Fig. 1B). Cluster 2 (*n* = 105 for 16S; 137 for 18S) largely represented microbial communities from the DCM in more stratified offshore sites (2 m–124 m; Fig. 1C), while Cluster 3 samples (*n* = 87 for 16S; 88 for 18S) were mainly from the aphotic zone (135 m–3,326 m), including offshore meso- to bathypelagic waters (Fig. 1D). There was some sample overlap between clusters, particularly for Clusters 1 and 2 (Fig. 2A and Fig. 3A). However, we distinguished communities in Clusters 2–3 from Cluster 1 based on the large proportion of samples confined to the open-ocean DCM (Cluster 2; 80%) and mesopelagic (Cluster 3; 98%) that reflect distinct habitats in the GOM.

Clusters aligned with global patterns, where depth often acts as a main driver of microbial composition in pelagic waters, reflecting vertical gradients in temperature, oxygen, nutrients, and organic matter (76–78). Indeed, we observed significant shifts in most abiotic factors across the three ecological depth clusters (Fig. S8). For example, temperature, pH, carbonate ion concentration, and aragonite saturation state all significantly decreased from Cluster 1 (photic zone) to Cluster 3 (aphotic zone), while most nutrients (NO_3_, PO_4_, and SiO_4_), DIC, and *p*CO₂ increased (Fig. S8). Other variables, such as oxygen, salinity, and NH_4_, remained relatively stable across clusters, whereas TA and NO_2_ peaked in Cluster 2 (DCM). Horizontal gradients, including proximity to shore and water mass properties, can also interact with depth to influence microbial community structure and ecological niches (79). In the photic zone, non-collinear factors used in microbial models exhibited spatial variability that often aligned with known environmental gradients in the GOM (80, 81). Temperature was higher offshore and in the southern GOM, while DIC concentrations were elevated in the southern GOM and onto the South Mexico Shelf (Fig. S9). Oxygen levels and salinity were lowest near the Mississippi River outflow, with higher salinities offshore (Fig. S9). Nutrient patterns were more variable: PO_4_ and NO_3_ were elevated near the Mississippi River and along coastal regions in the northern GOM, with NO_3_ also peaking in areas offshore in the southern GOM. Lastly, NH_4_ showed a patchier distribution, with higher values off the coast of Texas and Florida and generally lower concentrations offshore (Fig. S9).

Microbial biodiversity differed across the three ecological depth zones defined by our cluster analysis. Prokaryote richness and Shannon diversity index significantly increased from the photic to aphotic zones (Fig. 2B), consistent with the steady increase in diversity with absolute depth (Fig. S10). Higher prokaryotic diversity with depth has been observed in other ocean basins (76, 82, 83) and may be attributed to microbes utilizing a broad spectrum of sinking organic matter, exerting alternative metabolic strategies (redox reactions), and/or forming diverse trophic relationships with other organisms to exploit deep-water habitats (84). In contrast, protist diversity and richness peaked in the DCM and significantly decreased from the DCM to the aphotic zone (Fig. 3B), in line with overall trends based on absolute depth (Fig. S11). High protist diversity in the DCM may be related to the unique nature of these environments, where there is often a combination of elevated chlorophyll and nutrients, moderate light availability, and potentially reduced grazing pressure for certain protist taxa (85, 86). We measured CTD fluorescence on the cruise as a proxy for chlorophyll, which was highest on average in the offshore DCM (Fig. S8). Biodiversity remained relatively stable across lateral transects within the photic zone but showed greater variability in the DCM and aphotic zones (Fig. S12 and S13). For instance, in the aphotic zone, diversity declined from coastal Florida (27°N line) to regions near the Mississippi River outflow and then increased thereafter from the Brownsville to Cancun lines (Fig. S12).

Taxonomy also varied between clusters, consistent with observations made previously throughout the water column in parts of the GOM (32, 33, 35, 87, 88) and in other ocean basins (76, 77, 89). Among prokaryotes, the photic zone and DCM were dominated by common heterotrophic bacteria, such as SAR11, SAR86, and Flavobacteriales (Fig. 2C). Autotrophs within Synechococcales were also highly abundant in the photic zone (Fig. 2C), particularly *Prochlorococcus* and *Synechococcus* (Fig. S14), both genera known to dominate primary production in the GOM (90, 91). Prokaryotic communities shifted dramatically in the aphotic zone, with higher relative abundance of metabolically diverse taxa that are endemic to deeper waters (84, 88, 92), including nitrous oxide-reducing Marinimicrobia (SAR406), ammonia-oxidizing Nitrosopumilales, and sulfur-oxidizing Thiomicrospirales (Fig. 2C). These microbes use redox reactions to acquire energy in less oxygenated waters (84), such as those found in the mesopelagic zone (~200 m–1,000 m) in the GOM, where dissolved oxygen concentrations ranged from 117 to 175 µmol/kg (Fig. S8). Taxonomic differences between clusters also emerged at finer resolution. For instance, *Prochlorococcus* was more abundant in the DCM, while SAR11 clade II increased in relative abundance in the aphotic zone relative to other clades (Fig. S14), likely reflecting depth-based niche partitioning based on light, nutrients, or other resources (93, 94). High abundance of the SAR11 clade II in the mesopelagic has also been observed in the Pacific Ocean (84), indicating SAR11 may be associated with sinking particles.

Protist biodiversity was dominated by Dinophyceae, Syndiniales, Prymnesiophyceae, and Sagenista in the photic zone and DCM, transitioning to Radiolaria (Polycystinea and RAD-B) and Diplonemea in the aphotic zone (Fig. 3C). Dinophyceae and Prymnesiophyceae are widespread in pelagic waters, including in the GOM (31, 91), and occupy important functional roles as primary producers, grazers, mixotrophs, and symbionts in microbial food webs (2). Sagenista, a group of common, yet enigmatic heterotrophic protists (95, 96), was also abundant in the photic zone (Fig. 3C). Syndiniales were prevalent at all depths and sampling transects in the GOM (Fig. 3C). Often considered parasitic, the prevalence of Syndiniales likely reflects their wide host range, infection dynamics (e.g., rapid infection cycle and spore production), active (and passive) export on sinking particles, and depth-related niche partitioning (97, 98). We observed clade-level shifts within Syndiniales between depth zones that aligned with depth distributions reported in prior global sequencing surveys (97, 99). One clear example of this was a shift from Syndiniales Group-I Clades 1 and 4 in the photic zone to other clades, including Group-II Clade 7 and Group-I Clade 2 in the aphotic zone (Fig. S15). Radiolaria also varied between clusters, with certain members of Polycystinea (e.g., *Heliosphaera* and *Pterocorys*) becoming more relatively abundant from the DCM to aphotic zone (Fig. S15). While radiolarians remain largely uncultivated, they are key members of deep-ocean food webs, forming endosymbiotic relationships with other microorganisms and contributing to carbon and biogenic silica export (100). Finally, Diplonemea were a dominant protist group throughout the GOM in the aphotic zone (Fig. 3C), which is in line with recent 18S V9 surveys from other ocean basins (57) and supports their role as important heterotrophs in deep-ocean ecosystems (101).

### Group-specific environmental drivers in the photic zone

To identify environmental drivers of microbial abundance in the GOM, we applied Gaussian GAMs to log-transformed sequencing counts of dominant microbial groups (Fig. 2C and Fig. 3C). We focused modeling efforts on the photic zone, where there were more environmental variables that were non-collinear (Fig. S2). In contrast, Clusters 2 and 3 exhibited stronger collinearity among predictor variables, particularly between temperature and carbonate chemistry parameters (Fig. S2), and had fewer samples overall, limiting our ability to train models without overfitting (65). After filtering for collinearity (Spearman *r_s_* < |0.7| and VIF < 10), eight predictor variables were retained (Table 1): temperature, salinity, oxygen, NO_3_, PO_4_, NH_4_, pH (corrected), and DIC.

**TABLE 1:** Environmental factors used in microbial GAMs from the photic zone^*a*^

Parameter type	Factor	Values	VIF 16S (18S)
Hydrography	Temperature	20.83–30.12 (°C)	2.8 (2.4)
	Salinity	25.16–36.61 (psu)	7 (7.5)
	Oxygen	105.46–232.33 (µmol/kg)	3.1 (5)
Nutrients	Nitrate	0–6.16 (µmol/kg)	4.7 (4.2)
	Phosphate	0–0.85 (µmol/kg)	5.7 (6.2)
	Ammonium	0.12–2.37 (µmol/kg)	1.6 (1.7)
Carbonate chemistry	DIC	1,891.67–2,186.16 (µmol/kg)	9.1 (8.9)
	pH	7.88–8.16	5.2 (6.1)

^*a*^ Factors were grouped into parameter type and chosen for initial GAMs based on Spearman correlations (Fig. S2) and low variance inflation factors (VIF < 10) to mitigate collinearity among predictor variables. VIFs varied slightly between 16S and 18S (in parentheses) due to differences in sample size in the photic zone following clustering analysis (*n* = 270 for 16S; 233 for 18S). Triplicate samples were included in models. Data sets clustered similarly, as evidenced by a similar range in the predictor values, shown here for samples in the photic zone (Cluster 1) only. These initial factors were used to construct group-specific models. Temperature, salinity, and dissolved nutrients were log-transformed (pseudo-count of +0.1).

**Fig 4:**
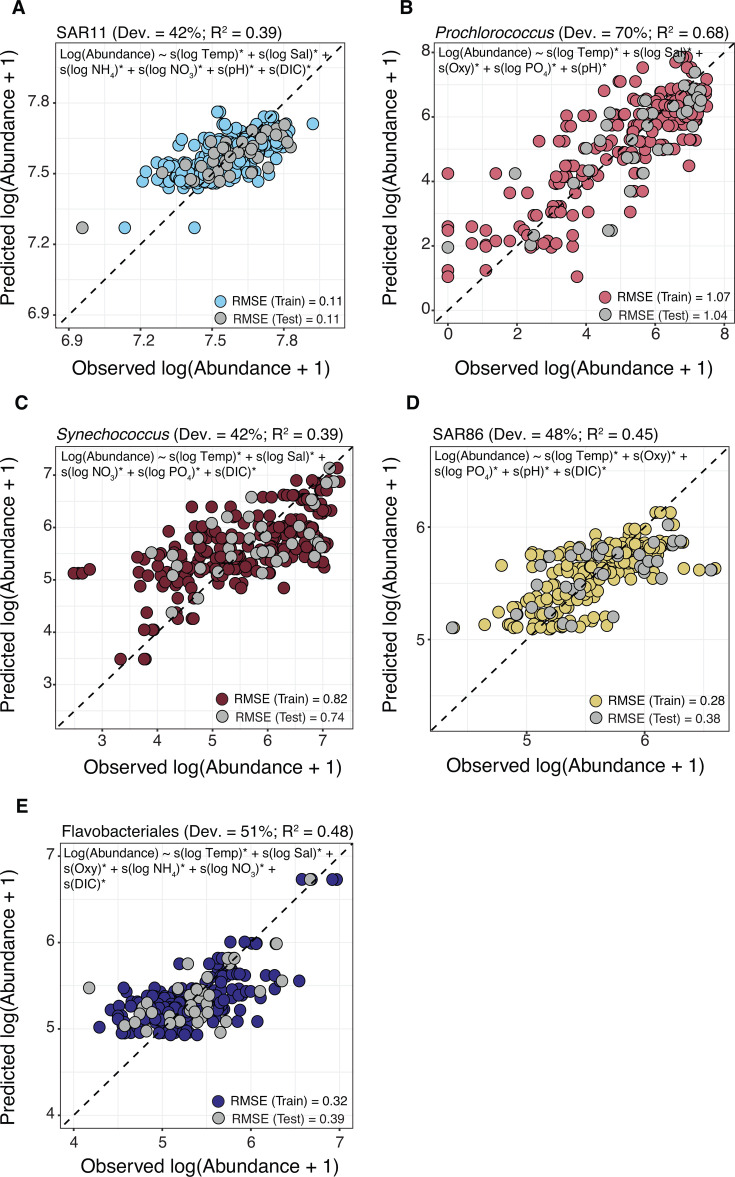
Model fit for major 16S groups. Observed vs predicted log-transformed abundance for the following groups that were modeled with a Gaussian GAM: SAR11 (**A**), *Prochlorococcus* (**B**), *Synechococcus* (**C**), SAR86 (**D**), and Flavobacteriales (**E**). Samples are colored by their use in the training (80%) or test set (20%; gray). Dashed lines indicate the 1:1 relationship between observed and predicted values. For each GAM, the final model formula, percent deviance explained, adjusted *R*^2^, and RMSE values are shown. Significant variables (asterisks in the model equation) were corrected for multiple comparisons (*P* < 0.05; Holm method). Final GAMs were selected with the training set using 10-fold cross-validation. A stratified partition based on sampling transect and abundance was used to split the samples into training and test sets to account for spatial variability in the photic zone. Abundances were log-transformed with a pseudo-count of +1 prior to model fitting.

**Fig 5:**
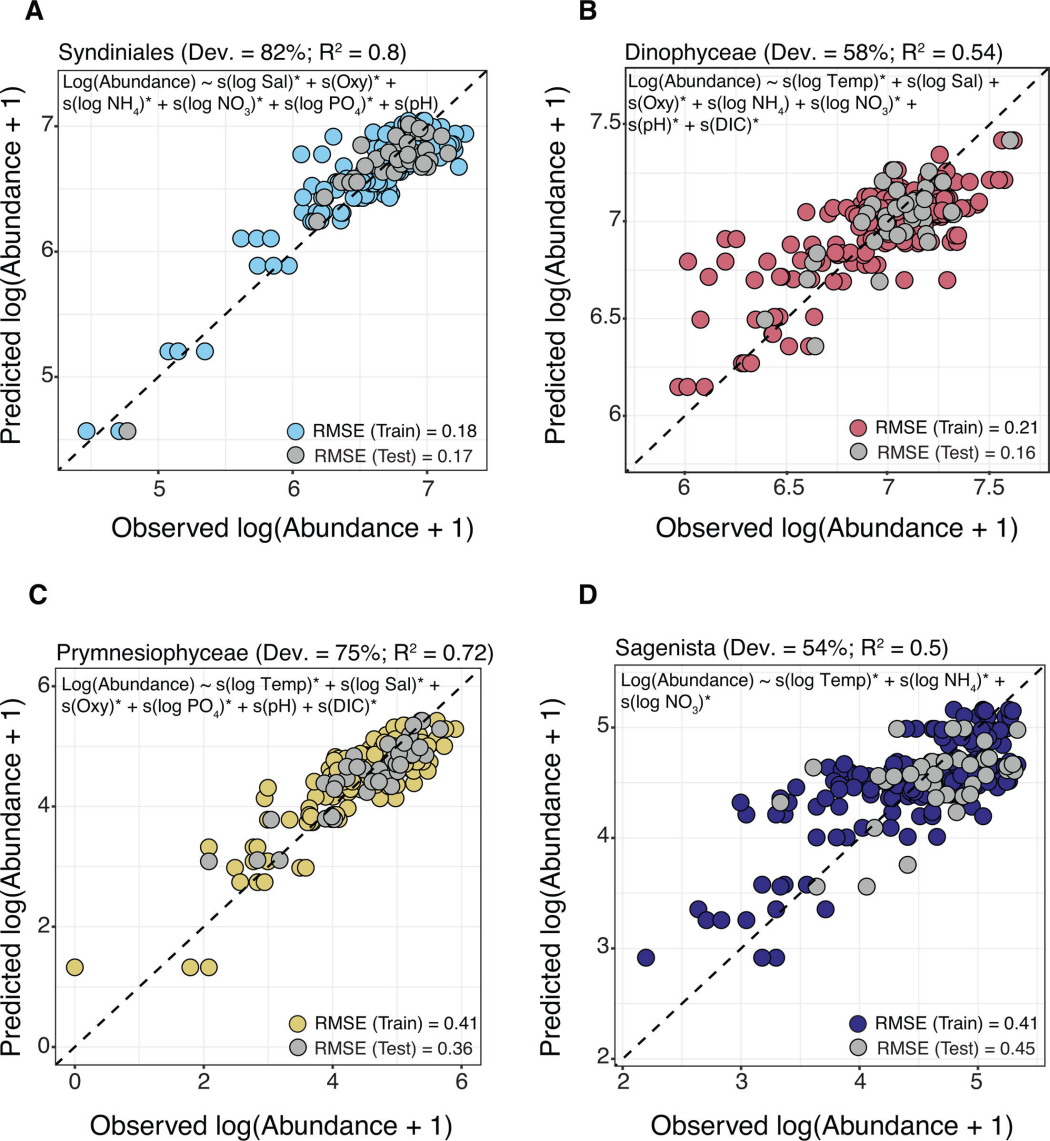
Model fit for major 18S groups. Observed vs predicted log-transformed abundance (pseudo-count of +1) for the following groups were modeled with a Gaussian GAM: Syndiniales (**A**), Dinophyceae (**B**), Prymnesiophyceae (**C**), and Sagenista (**D**). Samples are colored by their inclusion in training (80%) vs test sets (20%; in gray). Dashed lines indicate the 1:1 relationship between observed and predicted values. Final model formula, percent deviance explained, adjusted *R*^2^, and RMSE are shown for each group. Significant variables (asterisk in the model equation) were corrected for multiple comparisons (*P* < 0.05; Holm method). Final GAMs were selected with the training set using 10-fold cross-validation. A stratified partition based on sampling transect and abundance was used to split the samples into training and test sets to account for spatial variability.

To select environmental factors for final GAMs, we performed cross-validation on a training set (80%), employing a stratified data partition that was spatially aware and captured a range of log-abundance values and sampling transects in the data set. Across microbial taxa, GAMs trained on log abundance showed moderate to high explanatory power, with percent deviance explained ranging from 42 to 58% in some groups (adjusted *R*^2^ = 0.39–0.54) to over 70% (adjusted *R*^2^ = 0.68–0.8) in others (Table 2). These metrics, along with normality checks of model residuals and strong adherence to theoretical quantiles in Q–Q plots (Fig. S3), indicated that predictor variables selected via cross-validation were a good representation of microbial log abundance at the basin scale. In most models, RMSE values for training and test (20%) sets were aligned, and observed vs predicted values generally followed a 1:1 line (Fig. 4 and Fig. 5). However, model fits appeared tighter for major 18S groups compared to 16S groups overall, largely reflecting higher deviance explained among the 18S GAMs.

**TABLE 2:** Final GAMs for major microbial groups in the photic zone^*a*^

Group	Taxonomy	Final GAM	Dev. (%)	*R* ^2^
18S	Dinophyceae	s(logTemp)* + s(logSal) + s(Oxy)* + s(logNH_4_) + s(logNO_3_)* + s(pH)* + s(DIC)*	58	0.54
	Syndiniales	s(logSal)* + s(Oxy)* + s(logPO_4_)* + s(logNH_4_)* + s(logNO_3_)* + s(pH)	82	0.8
	Sagenista	s(logTemp)* + s(logNH_4_)* + s(logNO_3_)*	54	0.5
	Prymnesiophyceae	s(logTemp)* + s(logSal)* + s(Oxy)* + s(logPO_4_)* + s(pH) + s(DIC)*	75	0.72
16S	SAR11	s(logTemp)* + s(logSal)* + s(logNH_4_)* + s(logNO_3_)* + s(pH)* + s(DIC)*	42	0.39
	*Synechococcus*	s(logTemp)* + s(logSal)* + s(logPO_4_)* + s(logNO_3_)* + s(DIC)*	42	0.39
	*Prochlorococcus*	s(logTemp)* + s(logSal)* + s(Oxy)* + s(logPO_4_)* + s(pH)*	70	0.68
	SAR86	s(logTemp)* + s(Oxy)* + s(logPO_4_)* + s(pH)* + s(DIC)*	48	0.45
	Flavobacteriales	s(logTemp)* + s(logSal)* + s(Oxy)* + s(logNH_4_)* + s(logNO_3_)* + s(DIC)*	51	0.48

^*a*^ Protists were examined at the class level and prokaryotes at the order level. GAMs were also constructed for *Prochlorococcus* and *Synechococcus*. Models were run with a gaussian distribution and log-transformed abundances (pseudo-count of +1). Predictors were selected for final GAMs based on 10-fold cross-validation on a training set (80% of samples). Significant variables (*P* < 0.05; Holm correction) are shown in each model equation with an asterisk. Deviance explained (%) and adjusted *R*^2^ values of the final models are also shown. Temp, temperature (°C); Sal, salinity; Oxy, oxygen (µmol/kg); PO_4_, phosphate (µmol/kg); NO_3_, nitrate (µmol/kg); NH_4_, ammonium (µmol/kg); DIC, dissolved inorganic carbon (µmol/kg).

**Fig 6:**
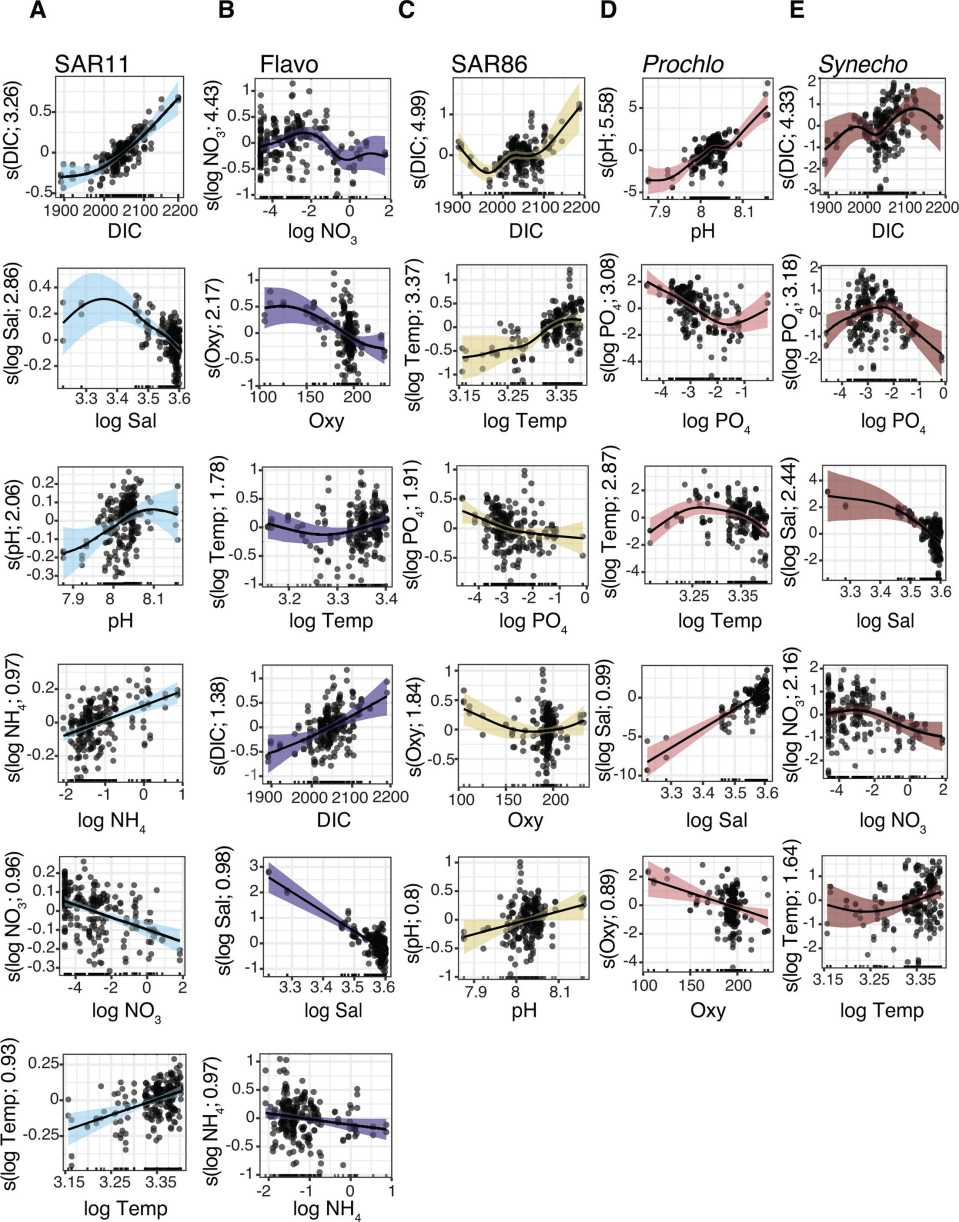
GAM partial effect plots of significant predictor variables for major 16S groups in the photic zone (Cluster 1). (**A–E**) Separate GAMs were constructed for the major groups as highlighted in Fig. 4. Shaded areas indicate 95% confidence intervals of the fitted line. Observed data points of each predictor variable are on the *x*-axis and the *y*-axis reflects the influence of the predictor on the response (log abundance) after smoothing. Groups are ordered left to right for visual clarity and not by relative abundance. Effective degrees of freedom (EDF) are shown in parentheses, and factors are ordered from high to low EDF from top to bottom for each group. A factor with a higher EDF indicates a more complex or nonlinear relationship between a predictor and response variable (EDF ~1 indicates a linear relationship). DIC, dissolved inorganic carbon; Temp, temperature; Sal, salinity; Oxy, oxygen; NO_3_, nitrate; PO_4_, phosphate; NH_4_, ammonium; *Prochlo*, *Prochlorococcus; Synecho*, *Synechococcus*; Flavo, Flavobacteriales. Certain variables were log-transformed prior to GAMs to correct for skewness. Full model equations are in Fig. 4 and Table 2.

**Fig 7:**
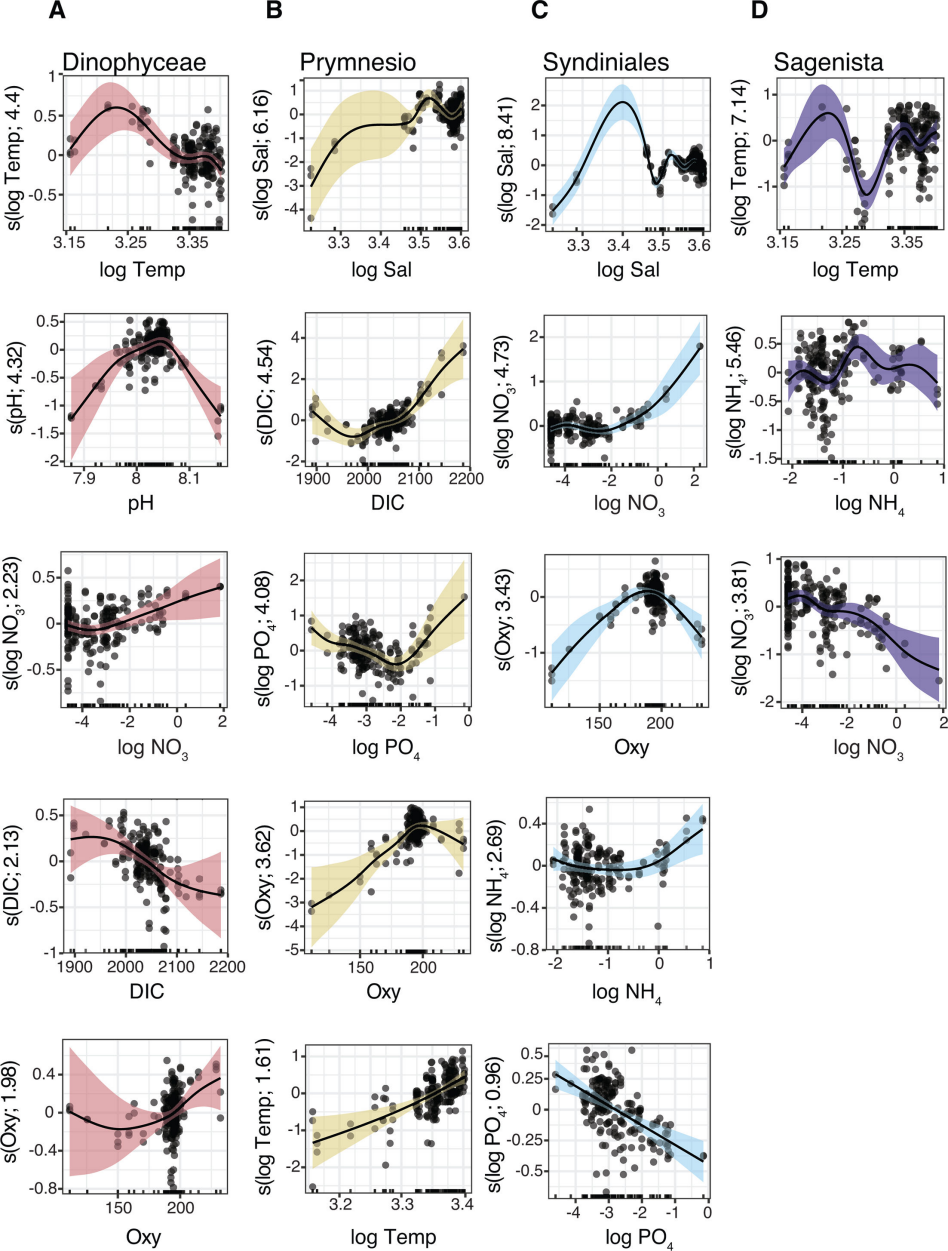
GAM partial effect plots of significant predictor variables for major 18S groups in the photic zone. (**A–D**) GAMs were constructed for the major class-level groups as shown in Fig. 5. Groups are ordered left to right for visual clarity and not by relative abundance (e.g., Syndiniales most abundant group but fewer significant predictors). Other details are the same as in Fig. 6, including ranking partial effects by EDF (top to bottom). DIC, dissolved inorganic carbon; Temp, temperature; Sal, salinity; Oxy, oxygen; NO_3_, nitrate; PO_4_, phosphate; NH_4_, ammonium; Prymnesio, Prymnesiophyceae. Certain variables were log-transformed prior to GAMs to correct for skewness. The full model equations are provided in Fig. 5 and in Table 2.

Group-specific partial effects revealed a range of linear and nonlinear associations between abiotic factors and microbial taxa in GOM surface waters (Fig. 6 and Fig. 7). Among major bacteria, SAR11 log abundance increased linearly with temperature and NH_4_, exhibited a nonlinear increase with DIC at higher concentrations, and was negatively associated with NO_3_ (Fig. 6A). There was also a dynamic unimodal relationship between SAR11 abundance and salinity, with abundance peaking at intermediate salinities (Fig. 6A). SAR11 abundance was positively associated with corrected pH, though with greater uncertainty at low and high values (Fig. 6A). SAR86 log abundance increased nonlinearly with temperature and DIC, decreased with PO_4_ and oxygen, and exhibited a positive (and weak) linear relationship with pH (Fig. 6C). Overall, these findings are consistent with the ecological roles of SAR11 and SAR86 as globally abundant bacteria that dominate stratified and low-nutrient surface oceans (76). Prior omics and flow cytometry studies have shown that future conditions, specifically warming (and increased stratification), may favor higher relative abundance and/or biomass of slower-growing (energy-efficient) bacteria such as SAR11 and SAR86 (102, 103). In general, warming is thought to enhance bacterial production, biomass, and respiration, though often at the expense of lower growth efficiency (18). Slower-growing bacteria have also been shown to preferentially use reduced nitrogen sources like NH_4_ (104), a pattern supported here for SAR11 (Fig. 6A). While not measured directly on this cruise, dissolved organic matter (DOM) availability and quality are known to influence bacterial communities (19). Therefore, it will be important to also consider shifts in DOM composition, driven by phytoplankton dynamics, temperature, or terrestrial inputs, which may amplify or moderate these abiotic associations.

*Prochlorococcus* log abundance was positively associated with salinity (linear) and pH (nonlinear), while negatively associated with oxygen (Fig. 6D), consistent with the dominance of this group in offshore waters (105). In addition, *Prochlorococcus* abundance was positively associated with PO_4_ at higher concentrations and displayed a unimodal relationship with temperature, where log abundance increased to a threshold and declined at the highest values (Fig. 6D). In contrast, *Synechococcus* showed more complex and nonlinear relationships with abiotic factors, including a unimodal relationship with PO₄, a stepwise increase with rising DIC, and a slight decline in log abundance at higher NO₃ concentrations (Fig. 6E). *Synechococcus* was positively associated with temperature at higher values, while salinity showed a gradual negative association (Fig. 6E). Laboratory and mesocosm studies have shown that elevated *p*CO_2_ and/or temperature can significantly increase biomass, growth rates, and photosynthetic capacity of *Synechococcus* but not *Prochlorococcus* (106, 107), while others have noted small or insignificant physiological impacts for either genus (108). In our samples, *Prochlorococcus* abundance increased to a threshold and declined at the highest temperatures, in line with recent modeling efforts of flow cytometry data from the tropical-subtropical Pacific Ocean that predict reductions in *Prochlorococcus* biomass (17%–51%) under future warming (109). Although other global models have predicted increased *Prochlorococcus* and *Synechococcus* biomass in warmer and more stratified conditions (110), our results from the GOM align with emerging field observations that imply more nuanced microbe-environment relationships, particularly in mixed communities (109). Furthermore, there is genomic evidence that S*ynechococcus* may be better equipped to tolerate future conditions, owing to its broader thermal range and stress-response capacity (111). Physiological studies and omics data sets rarely account for known ecotype-level variability within cyanobacteria (90, 112) or their potential to adapt to ocean change (17), areas of future research that will be critical for improving ecosystem and carbon models.

Syndiniales and Sagenista are ubiquitous and occupy important roles as protist parasites and heterotrophs in microbial food webs, respectively (96, 97). Yet, their environmental drivers remain poorly understood. For Syndiniales, log abundance declined linearly with PO_4_ and increased nonlinearly with NO_3_ and NH_4_ at higher nutrient concentrations (Fig. 7C). Syndiniales abundance also peaked at intermediate salinities and showed a unimodal relationship to oxygen, with highest abundance observed around ~175 µmol/kg–200 µmol/kg (Fig. 7C). While Syndiniales are often associated with low-oxygen environments in deeper waters or in oxygen minimum zones (99, 113), samples collected on this cruise were never below common thresholds for hypoxia (≤2 mg/L) even though seasonal hypoxia is common in shelf waters in the northern GOM (24). Nonetheless, Syndiniales were present at all sites and depths, implying broad oxygen tolerance that might be linked to clade-specific ecological preferences (99).

Sagenista log abundance exhibited a nonlinear association with temperature, with higher abundances at the extremes (Fig. 7D). This pattern may reflect variability among marine stramenopile (MAST) lineages, especially MAST-4B, 4C, and 7B, which made up the bulk of 18S sequences assigned to Sagenista in the photic zone (Fig. S15). Subclades within MAST-4 are known to be temperature-dependent, while MAST-7B has been associated with warm, offshore waters (95). The presence of lineage-specific thermal niches may have contributed to the dynamic temperature response we observed for this group at the class level. Sagenista log abundance was also negatively associated with NO_3_ and showed a modest increase with NH_4_ at intermediate concentrations (Fig. 7D). MAST cells are largely heterotrophic (114), and so their population dynamics likely rely on prey density and community composition (akin to parasite-host interactions). In general, the trophic activities of protist parasites and grazers remain poorly understood, in part because many of these taxa are not maintained in culture and are otherwise difficult to characterize in the field. Though the focus here was on biogeography and abiotic drivers, these data provide a strong foundation to investigate biological interactions (4). Leveraging amplicon data collected here and on future cruises, and integrating these data with co-occurrence analysis, targeted incubations, and culture experiments, will be important to further resolve microbial dynamics in the GOM.

Dinophyceae log abundance exhibited a nonlinear relationship with temperature, increasing initially but declining at the highest values (Fig. 7A). Abundance also showed a unimodal relationship with pH, peaking at intermediate values, as well as a weak negative association with DIC (opposite for oxygen) and a positive association with NO₃ (Fig. 7A). Prior culture and field studies have documented positive and negative responses of dinoflagellates to elevated *p*CO_2_ or temperature, with outcomes often varying at the species to strain level (20, 106, 115, 116). Many dinoflagellates, including genera observed in our samples (*Gymnodinium*, *Karenia*, and *Heterocapsa*; Fig. S15), are mixotrophic, capable of autotrophy and heterotrophy (117). Mixotrophic dinoflagellates display substantial flexibility in their physiologies, yet it remains unclear how changing temperature, pH, *p*CO_2_, or other factors will shift the balance between autotrophy and heterotrophy, trade-offs that could restructure trophic interactions and carbon cycling in future oceans (5, 117). Increased biomass of smaller picophytoplankton that is often predicted from global models may elicit increased rates of herbivory (118). Though shifts in prey composition may also favor other protist grazers, such as ciliates, that compete for similar resources (5). Along coastal regions, elevated nutrient inputs may further stimulate bloom events caused by mixotrophic (and often harmful) dinoflagellates (119), while diel vertical migration in stratified waters may help some species access deeper nutrient pools when surface concentrations are depleted (120). Ultimately, improving our ability to predict the response of dinoflagellates and other mixotrophs to changing conditions will depend on more resolved analyses of their functional and physiological traits through multi-omics studies, quantitative measurements (microscopy and cell sorting), and trait-based modeling (117).

Prymnesiophyceae log abundance was positively associated with DIC (nonlinear) and temperature (linear; Fig. 7B), in line with projections that smaller phytoplankton cells may respond more favorably to warming relative to larger taxa such as diatoms (8–10). Among other variables, log abundance showed a steady positive association with oxygen before stabilizing at higher values, a U-shaped relationship with PO_4_, and a nonlinear association with salinity, with high uncertainty at low salinities (Fig. 7B). Prymnesiophyceae in our surface samples were dominated by non-calcifying genera, such as *Chrysochromulina* and *Phaeocystis* (Fig. S15), consistent with a recent 18S rRNA survey in the southern GOM that reported high relative abundance of these genera in the mixed layer and DCM (35). Both genera are mixotrophic and may favor bacterial grazing under nutrient limitation to support bloom formation and/or increased production in oligotrophic waters (121, 122). In addition, *Chrysochromulina* has been shown to form photosymbiotic associations with nitrogen-fixing cyanobacteria of the UCYN-A clade (123), potentially enhancing its persistence when nutrient limited. By contrast, calcifying taxa, such as the coccolithophore *Gephyrocapsa* (formerly *Emiliania*) *huxleyi*, were less abundant in the data set (Fig. S15). This may reflect seasonality, as prior work has reported elevated *G. huxleyi* concentrations (~10⁴ cells per liter) in the southern GOM in springtime (124). Notably, however, an ASV assigned to *G. huxleyi* emerged as an indicator of more acidic surface conditions (see below), suggesting that despite its overall low relative abundance, this species may be important in the GOM as an indicator of ocean change.

While GAMs provided a baseline for identifying environmental correlates of microbial log abundance, several important caveats remain. First, our models were constructed from amplicon data collected during a specific time of the year (late summer/early fall), limiting our ability to resolve temporal changes. The GOM experiences strong seasonal and interannual variability in temperature, *p*CO₂, salinity, and dissolved nutrients that are driven by oceanographic features, such as the Loop Current, eddies, and riverine input, all of which can impact microbial communities (25, 125, 126). Second, although we captured nonlinear trends in our GAMs, uncertainty in the partial effects increased at the extremes for several predictor variables, where sparse data points occasionally produced strong curvature. These patterns should be interpreted more cautiously and highlight the importance of increasing spatial and temporal sampling to better constrain GAM predictions at environmental limits. To evaluate potential compositional bias in our amplicon data, we compared models using CLR-transformed and log-transformed read counts. CLR models often underperformed relative to log-based GAMs, indicating that log abundances were a better fit for our data set. More broadly, this underscores the importance of comparing multiple model types (e.g., GAMs vs GLMs) and data transformations. Third, our analyses focused on major taxonomic groups, but trends at the class or order level may obscure variability within these groups. Several microbial groups, including cyanobacteria and diverse protists, contain ecologically distinct clades, subclades, or ecotypes that will likely respond differently to environmental drivers. Finally, amplicon-based methods cannot resolve functional traits or physiological responses that may be important to consider in the context of changing conditions, such as those related to carbon or nutrient uptake, calcification, mixotrophy, or parasitism. Future efforts should aim to integrate single-cell genomics, metagenomics, and/or metatranscriptomics to better resolve clade-specific dynamics and link environmental change to microbial function (127). These approaches, when paired with sustained field observations in the GOM and elsewhere, will be essential for understanding how microbial communities will respond to changing conditions.

### Applying GAMs to expand spatial resolution of microbes in the GOM

**Fig 8:**
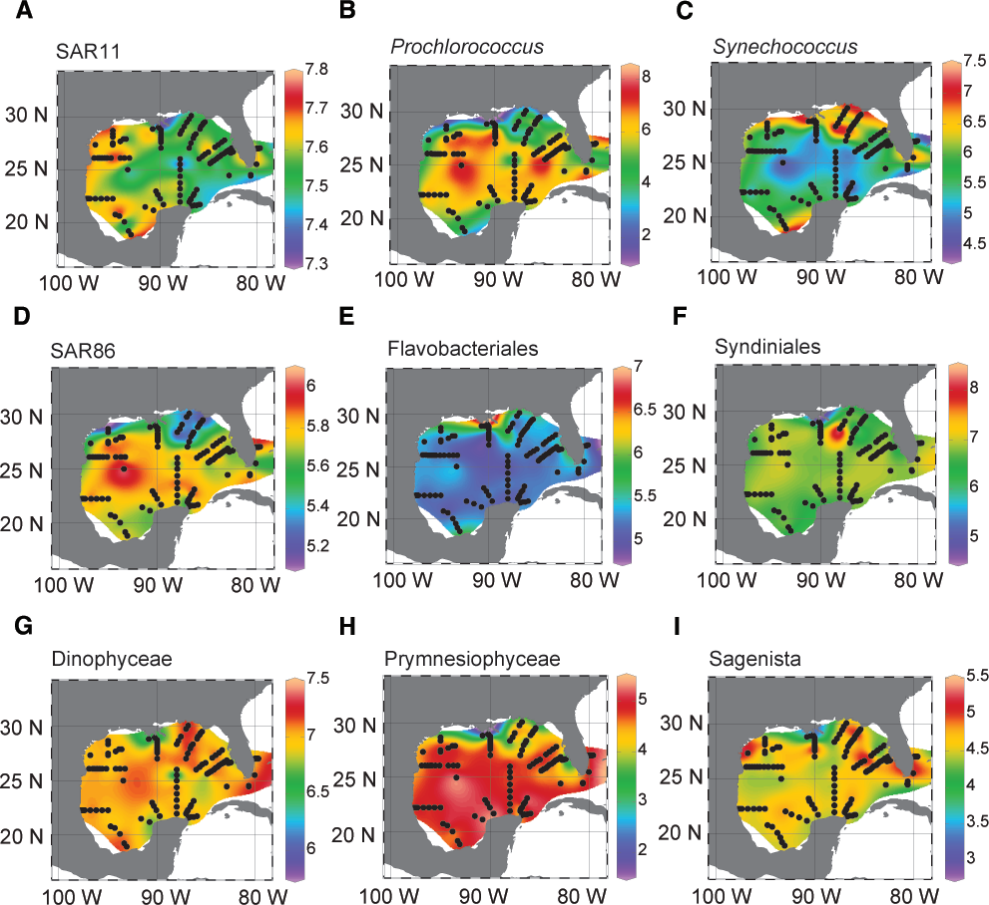
Predicting log abundance of microbial groups throughout GOM surface waters. Panels show predicted log-transformed abundance of major 16S (**A–E**) and 18S groups (**F–I**) at 135 sites, including 84 where DNA was not sampled. Predictions were made by applying final group-specific GAM equations that were determined from cross-validation. Model results have been interpolated using DIVA interpolation in Ocean Data View. Linear color scales were used for each respective group (right of panel), displaying gradients of low to high log abundance. Predictors were log-transformed when needed to match the final GAMs.

After selecting the best-fitting GAMs for each microbial group, we applied these models to predict log-transformed abundances at 135 surface stations sampled during GOMECC-4, including 84 sites where DNA was not collected (Fig. 8; Fig. S9). This extended our coverage of microbial biogeography across the GOM and allowed us to explore spatial abundance patterns beyond the sequenced data set. Among prokaryotes, SAR11 was widespread throughout the GOM, with predicted log-abundance values elevated in the west and southwest regions and peaking near the coast of Texas, along the East Mexico Shelf, and at sites in the Bay of Campeche (Fig. 8A). In comparison, SAR86 log abundance was predicted to be higher in offshore waters in the southern GOM (Fig. 8D), reflecting its positive association with elevated temperatures and DIC concentrations found in the model output for this group (Fig. 6E).

Cyanobacterial genera and Flavobacteriales were influenced by salinity and exhibited distinct spatial patterns consistent with known ecological niches (105, 128). *Prochlorococcus* was most abundant in offshore waters in the southern GOM (Fig. 8B), while *Synechococcus* peaked in coastal and shelf regions with lower salinity and elevated nutrients (Fig. 8C; Fig. S9), including areas influenced by the Mississippi River outflow. Flavobacteriales were similarly predicted to be most abundant in coastal waters near the Mississippi River (Fig. 8E), a pattern driven by negative associations with salinity (and oxygen) in the GAM results (Fig. 6B). The Mississippi River represents the largest freshwater and nutrient source to the GOM and can stimulate increased phytoplankton biomass, particularly during summer discharge events (125). Though diatoms were not broadly abundant across the GOM, their relative abundance peaked in the photic zone near the Mississippi River (Fig. 3C), possibly supporting increased abundance of fast-growing taxa such as Flavobacteriales that often associate with diatom blooms and can respond to pulses of labile organic matter (128).

Salinity is known to play a fundamental role in structuring microbial communities across the GOM (32, 129). The northern GOM is especially dynamic due to salinity-induced stratification driven by freshwater inputs from the Mississippi–Atchafalaya system and large-scale climatic variability (e.g., ENSO). These drivers shape vertical and horizontal gradients in nutrient supply, light availability, and water column structure, in turn influencing microbial distributions (34, 129, 130). Although our analysis focused on basin-scale trends rather than localized responses, many of the relationships recovered in our models likely reflect this underlying salinity-driven heterogeneity. Strong surface salinity gradients, particularly in the northern GOM, may have also enhanced our ability to detect these associations (129). While such salinity variability is especially pronounced in the GOM and may not directly translate to more oceanic systems, similar dynamics are likely at play in other coastal and estuarine-influenced waters and are important to consider when determining microbial dynamics.

Syndiniales and Dinophyceae were widespread across the GOM (Fig. 8F and G), with Dinophyceae predicted to be most abundant in the southern GOM, off the coast of Panama City and Pensacola (Florida), and along the East Mexico Shelf (Fig. 8G). Prymnesiophyceae was most abundant in offshore waters and in all regions of the southern GOM (Fig. 8H). In contrast, Sagenista exhibited highly variable spatial patterns, with patches of elevated predicted log abundance across both coastal and offshore regions (Fig. 8I). This spatial heterogeneity may reflect the diversity of MAST lineages within Sagenista, where cells occupy distinct environmental or geographic niches (95). Further investigation is warranted to explore MAST dynamics in the GOM at more resolved taxonomic levels. Overall, spatial predictions based on GAMs provided ecologically realistic patterns for major protist and prokaryote groups, extending our insight into their distribution in undersampled areas of the GOM and offering a foundation for future biological sampling.

### Indicator analysis reveals microbial signatures of OA

**Fig 9:**
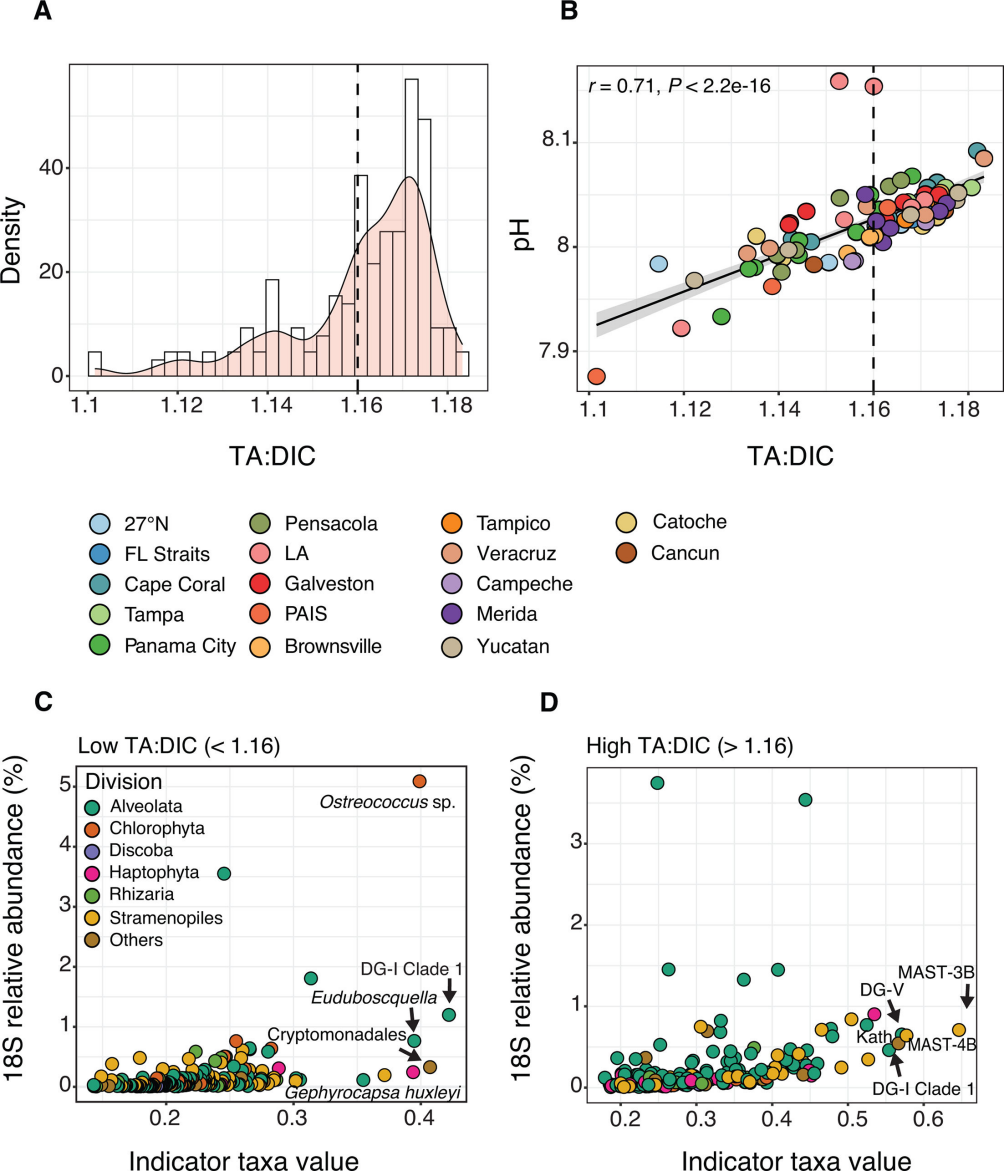
Protist indicator taxa of different OA conditions in the photic zone. (**A**) Histogram of 18S sample distribution in the photic zone based on TA:DIC ratios. The TA:DIC ratio indicates the ocean’s buffering capacity to absorb and buffer against increased atmospheric CO_2_. (**B**) Values of *in situ* pH vs TA:DIC in the photic zone, with samples colored by transect. Pearson correlation between variables is shown, with a 95% CI. The dotted lines in panels A-B indicate the manual cutoff used for indicator analysis: low TA:DIC < 1.16 vs high TA:DIC > 1.16. (**C and D**) Indicator values vs mean relative abundance (%) for protist ASVs in the photic zone that were significant in the indicator analysis (*P* < 0.05; Benjamini-Hochberg correction) in samples with either low (**C**) or high TA:DIC (**D**). Indicator analysis tests whether taxa occur more often in particular sample groups than would be expected by chance. Protist ASVs are colored by division, and the top 5 ASVs with the highest indicator values are labeled in each panel and identified to their lowest possible taxonomic assignment (via the PR2 database). DG, Dino-Group; Kath, Kathablepharidida. See Table S5 for a full list of 18S indicator ASVs.

Understanding how microbial communities respond to changing carbonate chemistry remains important to assess the impacts of OA on marine ecosystems (5–7). To investigate potential microbial indicators of OA conditions in the GOM, we focused on photic zone DNA samples collected on GOMECC-4 (Cluster 1) and examined ASVs associated with variation in the TA:DIC ratio. This ratio is a widely used proxy for carbonate buffering capacity in seawater, with lower values indicating reduced resistance to acidification (64). In our data set, TA:DIC ratios in the photic zone ranged from 1.1 to 1.2, were positively correlated with surface water pH (Pearson *r* = 0.71, *P* < 0.01), and showed no significant pattern across sampling transects, implying this gradient was independent of location (Fig. 9A and B).

We grouped samples into low (<1.16) and high (>1.16) TA:DIC categories based on histogram distributions (Fig. 9A) and applied indicator analysis to identify taxa more likely to occur in one category versus the other than would be expected by chance. This method estimates the strength and statistical significance of associations between ASVs and predefined groups, adjusting for multiple comparisons (63). In total, 180 and 244 protist ASVs were identified as significant indicators of low and high TA:DIC categories, respectively (*P* < 0.05; Table S5). Indicator ASVs spanned a range of taxa, though several emerged as particularly informative (Fig. 9C and D). Among low TA:DIC samples that represent more acidified waters, ASVs with high indicator values (>0.38) included *Ostreococcus* sp., the most relatively abundant indicator in the photic zone (~5% on average), as well as ASVs assigned to *Gephyrocapsa huxleyi*, Cryptomonadales, *Euduboscquella* (Syndiniales), and Syndiniales Group-I Clade 1 (Fig. 9C). In contrast, samples with high TA:DIC ratios were associated with ASVs assigned to MAST-3B (Opalozoa) and 4B (Sagenista), Kathablepharidida, and Syndiniales Group-V, each with strong indicator values (>0.54; Fig. 9D). Interestingly, no ASVs in the 16S data set were significantly associated with high or low TA:DIC after correcting for multiple comparisons (*P* > 0.05; Table S5). This suggests that in our data set there was no statistical evidence to support specific prokaryotic indicators of TA:DIC. While we cannot draw broad conclusions from this alone, prior studies have suggested that prokaryotes may be less sensitive to ocean change compared to protists (131), which may partly explain the lack of detectable associations here.

Among protist taxa identified in our indicator analysis, both *Ostreococcus* and *G. huxleyi* were more prevalent in less buffered surface waters (i.e., lower TA:DIC ratios; Fig. 9C). These widespread taxa are important to global biogeochemical cycles (132), with *G. huxleyi* serving as a major marine calcifier and contributor to calcium carbonate flux (133). A recent 18S V9 survey in the southern GOM found *Ostreococcus* to be the only genus with significantly different relative abundance between upwelling and downwelling conditions in the DCM, and when comparing samples from the DCM to the mixed layer (35), which authors suggest may make this species indicative of NO_3_ flux in the upper water column. Our results also point to *Ostreococcus* as a candidate microbial indicator of carbonate chemistry in GOM surface waters. Calcifying organisms, such as *G. huxleyi*, are often considered to be sensitive to elevated *p*CO₂ and reduced pH, with reported negative impacts on calcification and growth rates (13, 14). However, these responses can be strain-specific (16), and long-term culture studies have shown that both *G. huxleyi* and *Ostreococcus* can exhibit adaptive capacity to OA (134, 135). Overall, these findings support the potential for pico- to nanoplankton to serve as indicators of environmental change in the GOM and highlight the need to further investigate their ecology and physiology in this ecosystem over time and space.

### Conclusions

By building GAMs with amplicon data from the GOM, we captured linear and nonlinear associations between environmental factors and log abundance of microbial groups, patterns that would have been overlooked using linear models alone. These models were applied to all cruise sites, including those not sampled for DNA, to expand predictions of microbial biogeography at the basin scale. Importantly, these findings reflect a single cruise in summer–fall, and thus, while results offer valuable baseline measurements, there remains a need for additional temporal sampling. Together, this approach provided new insights into the distribution and environmental drivers of microbes in the dynamic GOM. Samples clustered into three ecological depth zones, and GAMs applied to the photic zone revealed group-specific responses to non-collinear predictors. Temperature and DIC were positively associated with SAR11 and SAR86, consistent with prior findings that suggest warming and increased carbon availability may favor slower-growing bacterial groups. *Prochlorococcus* and *Synechococcus* showed divergent relationships with salinity, temperature, and nutrients, reflecting known niche differentiation across coastal-offshore environments and implying potential differences in their thermal range. Environmental drivers for Syndiniales and Sagenista, two widespread but poorly characterized protist groups, varied and included combinations of salinity, oxygen, and nitrogen sources. At finer taxonomic resolution, several 18S ASVs (e.g., *Ostreococcus* and *G. huxleyi*) were more abundant in surface waters with lower TA:DIC ratios and may act as candidate indicators of OA to further verify.

Future changes to ocean ecosystems, which include acidification, warming, deoxygenation, and nutrient runoff, are expected to elicit shifts in microbial biogeography, physiology, and interactions (6). In turn, this may lead to a reshaping of ecosystem functioning, with implications for carbon export, nutrient cycling, and trophic dynamics (5, 8). While some microbes may exhibit adaptive capacity to changing conditions in culture, the relevance of this adaptation in natural, mixed communities remains uncertain (17). To better understand microbes and their responses to environmental factors, there is a need for sustained, spatially resolved sampling that integrates molecular data with environmental and oceanographic observations. Despite measurable increases in surface *p*CO₂ in the GOM, long-term microbial monitoring efforts remain limited compared to other ocean basins (22). Expanding biological sampling within existing programs like GOMECC, and incorporating tools such as remote sensing, autonomous platforms, and coupled ecosystem-climate models will be essential (33). These efforts will improve our ability to forecast microbial dynamics and biodiversity under future scenarios and support decision-making by resource managers, policymakers, and coastal communities who depend on GOM ecosystems.

## Data Availability

Code and files needed to reproduce results and figures are available on GitHub (https://github.com/aomlomics/gomecc) and have been archived on Zenodo (https://zenodo.org/records/13102580). All 18S and 16S sequences have been published at the National Center for Biotechnology Information (NCBI)’s Sequence Read Archive and BioSample database and are available with BioProject accession number PRJNA887898. Species counts generated from this study have been published on the Ocean Biodiversity Information System (OBIS) and the Global Biodiversity Information Facility (GBIF) at https://ipt-obis.gbif.us/resource?r=noaa-aoml-gomecc4 and https://doi.org/10.15468/sm6fpz, respectively. Species counts are also included among all marine biological occurrence data managed by OBIS-USA, archived by the National Centers for Environmental Information (NCEI) at https://www.ncei.noaa.gov/archive/accession/0250940. Environmental measurements from the Niskin bottles and CTD profiles are also available at NCEI at https://doi.org/10.25921/4twf-pp50 and https://doi.org/10.25921/04h7-gv36, respectively. A cruise report detailing all the sampling and analyzing procedures during GOMECC-4 is available (41).

## SUPPLEMENTAL MATERIAL

Supplemental material aem.01889-25-s0001.pdf Supplemental methods, Fig. S1 to S15, and legends for Tables S1 to S5.

Table S1 aem.01889-25-s0002.xlsx Metadata at each site and depth that coincided with DNA sampling.

Table S2 aem.01889-25-s0003.xlsx Functional assignments for all 18S and 16S sequences identified in DNA samples.

Table S3 aem.01889-25-s0004.xlsx Spearman rank correlations between environmental factors for 16S and 18S data sets and the three clusters.

Table S4 aem.01889-25-s0005.xlsx Model comparisons of major microbial groups.

Table S5 aem.01889-25-s0006.xlsx Indicator analysis results for 16S and 18S samples.

ASM does not own the copyrights to Supplemental Material that may be linked to, or accessed through, an article. The authors have granted ASM a non-exclusive, world-wide license to publish the Supplemental Material files. Please contact the corresponding author directly for reuse.
