# Supplementary material for: Microbial community dynamics over large spatial and environmental gradients in a subtropical ocean basin
Source: Appl Environ Microbiol. 2026 Jan 22;92(2):e01889-25. doi: 10.1128/aem.01889-25 (PMC12915303; doi:10.1128/aem.01889-25)
Supplement: Supplemental material — Supplemental methods, Fig. S1 to S15, and legends for Tables S1 to S5. [file aem.01889-25-s0001.pdf]

Supplemental material

Microbial community dynamics over large spatial and environmental gradients in a subtropical ocean basin

Sean Anderson *et al.*

Corresponding authors: Sean Anderson and Luke Thompson
Emails: seanceltics34@gmail.com and luke.thompson@noaa.gov

This SI file includes:

Additional methods and references
Figures S1 to S15
Legends for Tables S1 to S5

Other supplemental materials for this manuscript included in separate files:

Table S1
Table S2
Table S3
Table S4
Table S5

Additional methods:

Discrete chemical data collection and processing

Discrete samples for water chemistry were collected from all 141 sites, which included 51 sites sampled for DNA. Blanks and quality control samples were considered for each discrete chemical parameter. Dissolved oxygen concentration was estimated from water samples (125 ml) using an automated oxygen titrator with amperometric end-point detection (1). Nutrient samples were collected from Niskin bottles into 50-ml acid washed bottles. Dissolved nutrients (NO_3, NO_2, NH_4, PO_4, and SiO_4) were measured on board using an automated continuous flow analytical system with colorimetric detection ((2); SEAL Analytical). Samples for DIC were collected from Niskin bottles into 294-ml borosilicate glass bottles, sealed with glass stoppers, and stored for 12 h at room temperature. DIC samples were analyzed on the ship using two analytical systems, each consisting of a coulometer (CM5017, UIC Inc.) coupled with a Dissolved Inorganic Carbon Extractor (3).

Samples for total alkalinity (TA) were collected from Niskin bottles into 500-ml collection bottles, preserved with a mercuric chloride solution, and kept in a water bath at 22 ºC for 1 h prior to analysis. TA measurements were made using a two-titration system, consisting of a Metrohm 765 or 665 Dosimat Titrator and Orion 720A or 2-Star pH meter (4). Samples for pCO_2 were drawn from Niskin bottles into 500-ml glass bottles, preserved with mercuric chloride, and stored at room temperature for 8 h before analysis. Details on the system used to measure pCO_2 are described in (5) and include equilibrating each sample with a constantly circulating gas phase. Lastly, for pH analysis, samples were collected from Niskin bottles into 10-cm (~30 ml) glass cylindrical optical cells and analyzed on an Agilent 8453 spectrophotometer with a custom-made temperature-controlled cell holder (6).

Functional assignments

Protist ASVs were manually assigned to different functional groups based on 18S V9 functional annotations made in (7) and available via an archived repository (https://doi.org/10.5281/zenodo.3768950). These annotations were previously applied to Tara Ocean protistan communities. The following groups were included for 18S assignments: autotrophic protists, heterotrophic protists, mixotrophic protists, parasites, photosymbionts, and other protists. Mixotrophic protists were further categorized as being constitutive mixotrophs (CM) that inherently have chloroplasts and endosymbiotic specialist non-constitutive mixotrophs (eSNCM) that harbor endosymbionts to support growth (8, 9). Some dinoflagellates were assigned as heterotrophs or symbionts, but they were largely characterized as mixotrophs in the dataset (8). We recognize that many protists likely exhibit mixotrophy in some capacity, and so, our functional annotation of this group may be underrepresented. Other protists represented

higher level taxonomic groups (domain or supergroup) that were unassigned at lower levels. Bacteria and Archaea were categorized functionally as heterotrophic or autotrophic. Refer to Table S2 for a complete list of ASV functional assignments inferred in the dataset.

References:

1. Langdon C. 2010. Determination of dissolved oxygen in seawater by Winkler titration using the amperometric technique.

2. Zhang J-Z, Berberian GA. 1997. Determination of dissolved silicate in estuarine and coastal waters by gas segmented continuous flow colorimetric analysis. Methods for the determination of chemical substances in marine and estuarine environmental matrices 366–360.

3. Johnson KM, King AE, Sieburth JM. 1985. Coulometric TCO_2 analyses for marine studies; an introduction. Mar Chem 16:61–82.

4. Dickson A, Sabine C, Christian J. 2007. Guide to best practices for ocean CO_2 measurements.

5. Wanninkhof R, Thoning K. 1993. Measurement of fugacity of CO_2 in surface water using continuous and discrete sampling methods. Mar Chem 44:189–204.

6. Byrne RH, Kump LR, Cantrell KJ. 1988. The influence of temperature and pH on trace metal speciation in seawater. Mar Chem 25:163–181.

7. Cordier T, Angeles IB, Henry N, Lejzerowicz F, Berney C, Morard R, Brandt A, Cambon-Bonavita M-A, Guidi L, Lombard F, Arbizu PM, Massana R, Orejas C, Poulain J, Smith CR, Wincker P, Arnaud-Haond S, Gooday AJ, de Vargas C, Pawlowski J. 2022. Patterns of eukaryotic diversity from the surface to the deep-ocean sediment. Sci Adv 8:eabj9309.

8. Mitra A, Caron DA, Faure E, Flynn KJ, Leles SG, Hansen PJ, McManus GB, Not F, do Rosario Gomes H, Santoferrara LF, Stoecker DK, Tillmann U. 2023. The Mixoplankton Database (MDB): Diversity of photo-phago-trophic plankton in form, function, and distribution across the global ocean. J Eukaryot Microbiol 70:e12972.

9. Millette NC, da Costa M, Mora JW, Gast RJ. 2021. Temporal and spatial variability of phytoplankton and mixotrophs in a temperate estuary. Mar Ecol Prog Ser 677:17–31.

Supplemental figures:

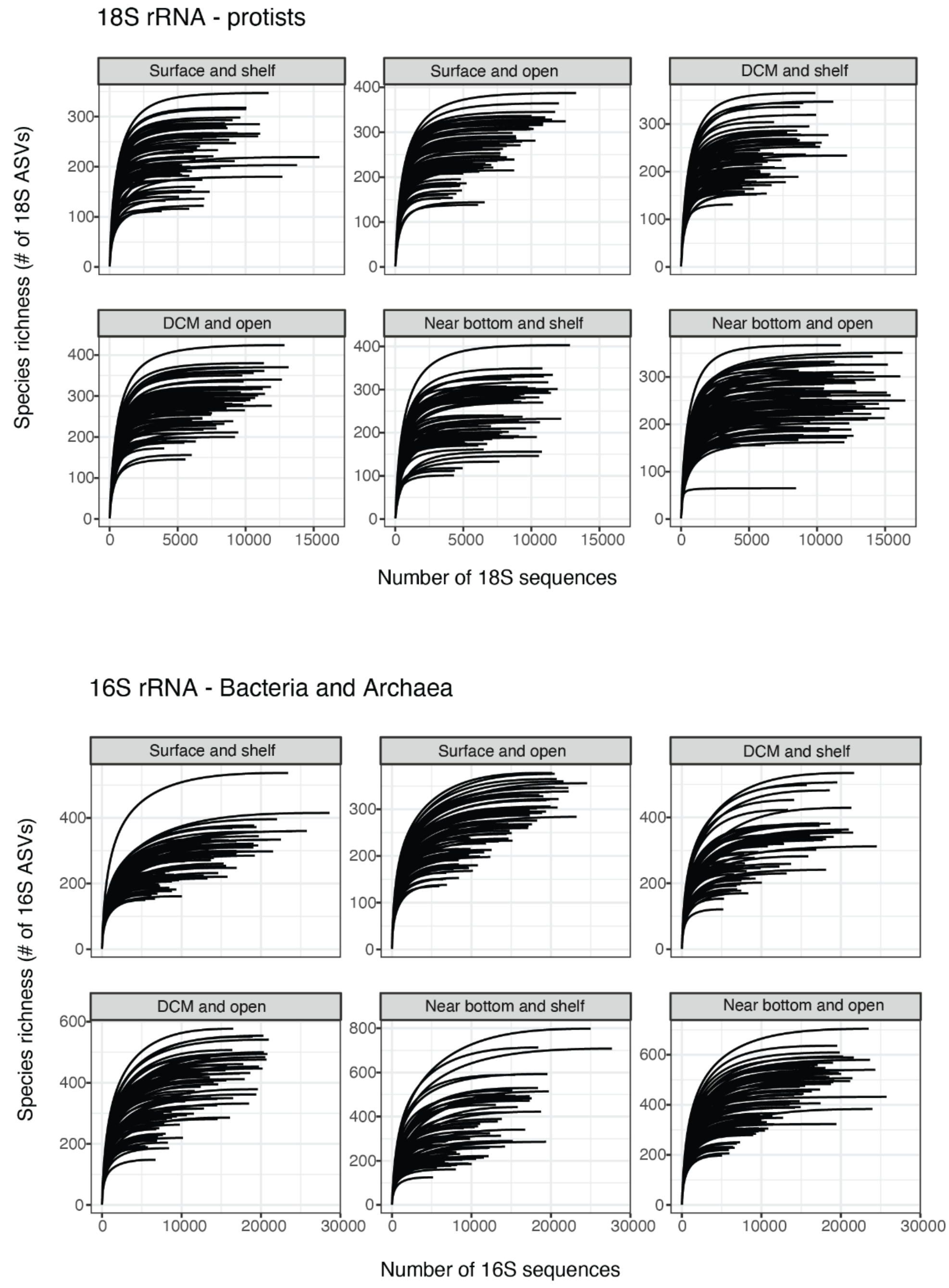

Figure S1: Rarefaction curves of 18S (top) and 16S (bottom) species richness (# of ASVs) vs. sequence read counts across all samples and faceted by categorical sampling depth (surface, deep chlorophyll maximum or DCM, and near bottom) and position of samples on the continental shelf vs. open ocean. Curves were estimated using a step size of 100.

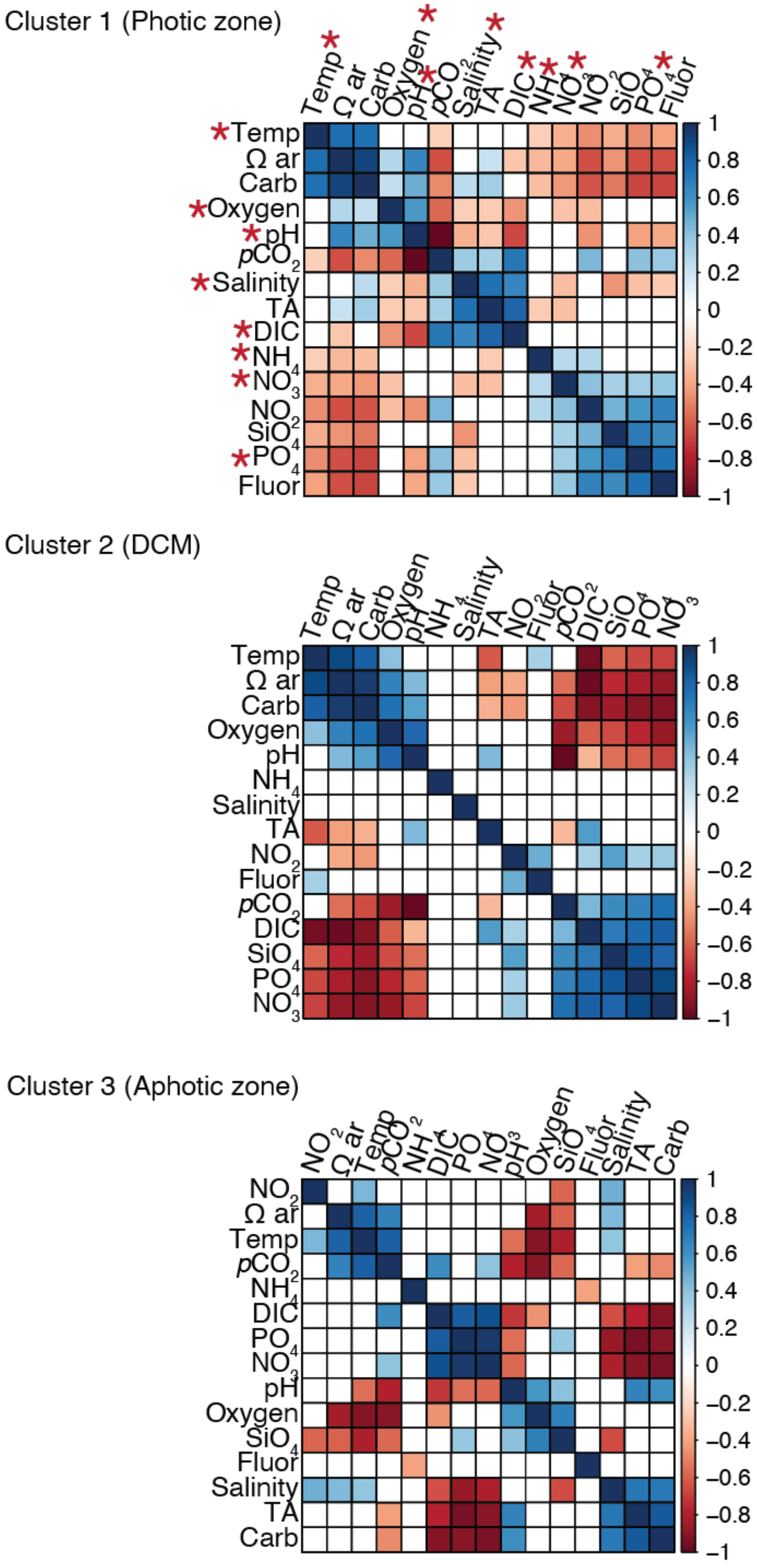

Cluster 1 (Photic zone)
Temp
Ω ar
Carb
Oxygen
pH
pCO2
Salinity
TA
DIC
NH4
NO3
NO2
SiO4
PO4
Fluor
1
0.8
0.6
0.4
0.2
0
−0.2
−0.4
−0.6
−0.8
−1
Cluster 2 (DCM)
Temp
Ω ar
Carb
Oxygen
pH
NH4
Salinity
TA
NO2
Fluor
pCO2
DIC
SiO4
PO4
NO3
Cluster 3 (Aphotic zone)
NO2
Ω ar
Temp
pCO2
NH4
DIC
PO4
NO3
pH
Oxygen
SiO4
Fluor
Salinity
TA
Carb

Figure S2: Spearman correlations between environmental variables in Clusters 1–3 associated with the 18S dataset. Only significant correlations are shown ($P < 0.05$), with the sign of the correlation indicated by a red (negative) to blue (positive) gradient. Stronger correlations are indicated by darker colors, while white boxes indicate no significance. In this case, we performed correlation analysis to select variables for initial models that had low collinearity, and so associations that were not significant or had Spearman $r_s < |0.7|$ were selected for variance inflation factor (VIF) analysis to confirm low multicollinearity (VIF < 10). Models focused on Cluster 1, with chosen parameters indicated by red asterisks. Models were not constructed for Clusters 2 and 3, as more variables were strongly collinear with each other and there were fewer samples overall. Temp = temperature (°C); Oxy = oxygen (µmol kg^{-1}); Sal = salinity; Fluor = fluorescence; PO_4 = phosphate (µmol kg^{-1}); SiO_4 = silicate (µmol kg^{-1}); NO_3 = nitrate (µmol kg^{-1}); NO_2 = nitrite (µmol kg^{-1}); NH_4 = ammonium (µmol kg^{-1}); DIC = dissolved inorganic carbon (µmol kg^{-1}); TA = total alkalinity (µmol kg^{-1}); Carb = carbonate (µmol kg^{-1}); pCO$_2$ = partial pressure of CO_2 (µmol kg^{-1}); Ω ar = aragonite saturation state.

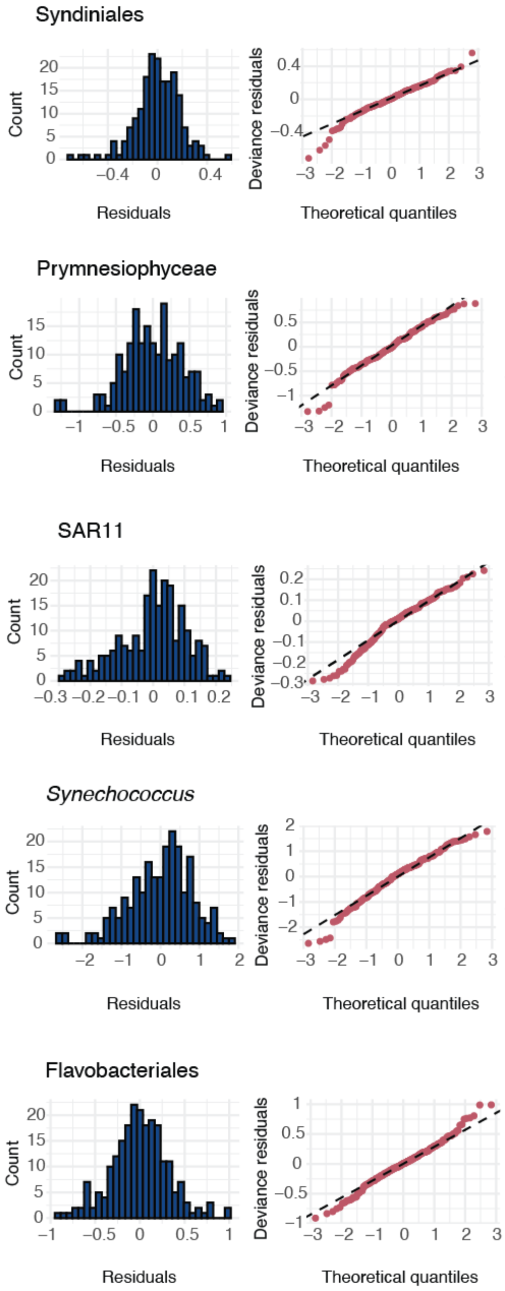

Figure S3: Diagnostic plots for group-specific Gaussian GAMs (with log abundance) from samples collected in the photic zone. Results for each of the major 18S and 16S groups are shown. The histograms display the distribution of the residuals, which is roughly symmetrical across groups and indicates the residuals were approximately normally distributed. The Q–Q (quantile-quantile) plots compare the observed quantiles of the deviance residuals to the theoretical quantiles of a normal distribution.

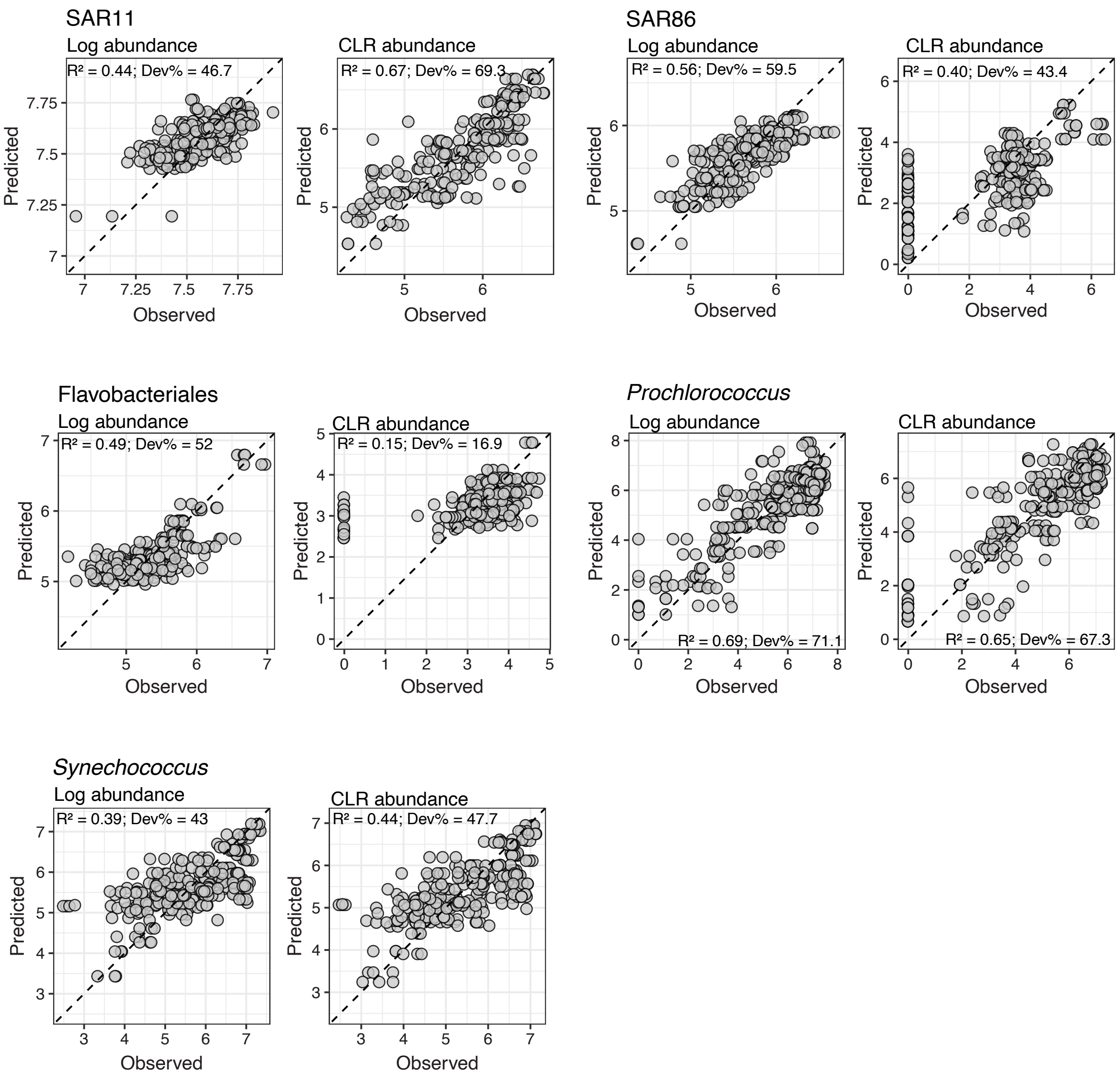

Figure S4: Predicted vs. observed abundance for major 16S groups in the photic zone that were modeled with a Gaussian generalized additive model (GAM) based on log (left panel) or centered log-ratio (CLR) transformation (right panel). For each GAM, percent deviance explained and adjusted R^2 values are shown, with dashed lines indicating the 1:1 relationship between observed and predicted abundance. Abundances had a pseudo-count of + 1 prior to modelling. All non-collinear environmental factors were considered for testing. See Table S4 for model AIC values.

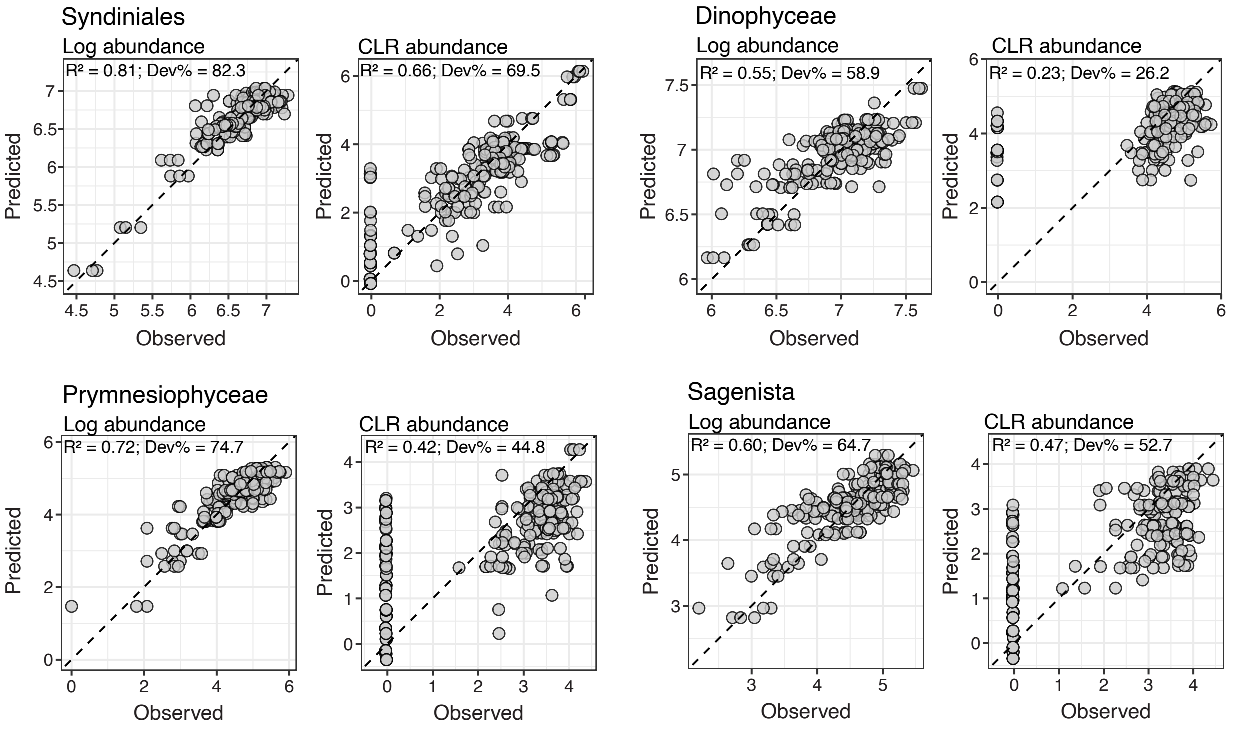

Figure S5: Predicted vs. observed abundance for major 18S groups in the photic zone that were modeled with a Gaussian GAM based on log (left panel) or CLR transformation (right panel). For each GAM, percent deviance explained and adjusted R^2 values are shown, with dashed lines indicating the 1:1 relationship between observed and predicted abundance. Other details are the same as in Fig. S4. See Table S4 for model AIC values.

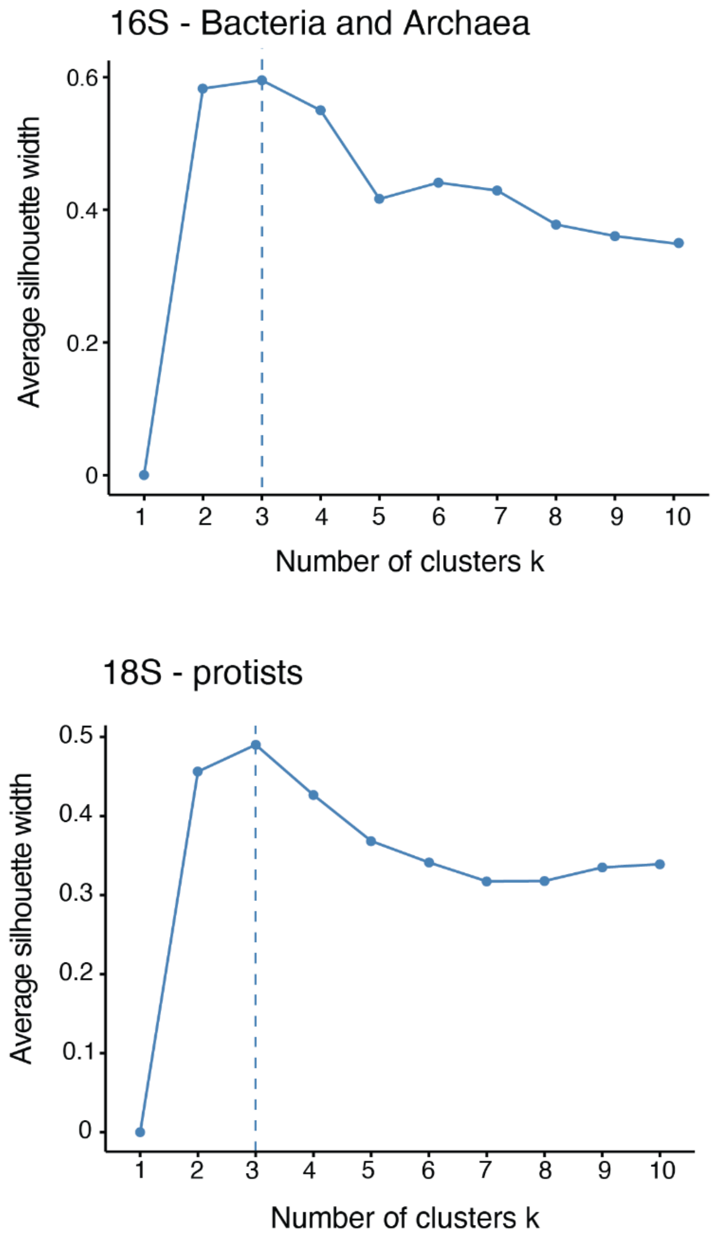

Figure S6: Average silhouette widths vs. number of clusters based on hierarchical clustering of 16S (top) and 18S (bottom) Aitchison distance matrices. The optimal number of clusters (three) is indicated by the dotted line and is the same for both datasets.

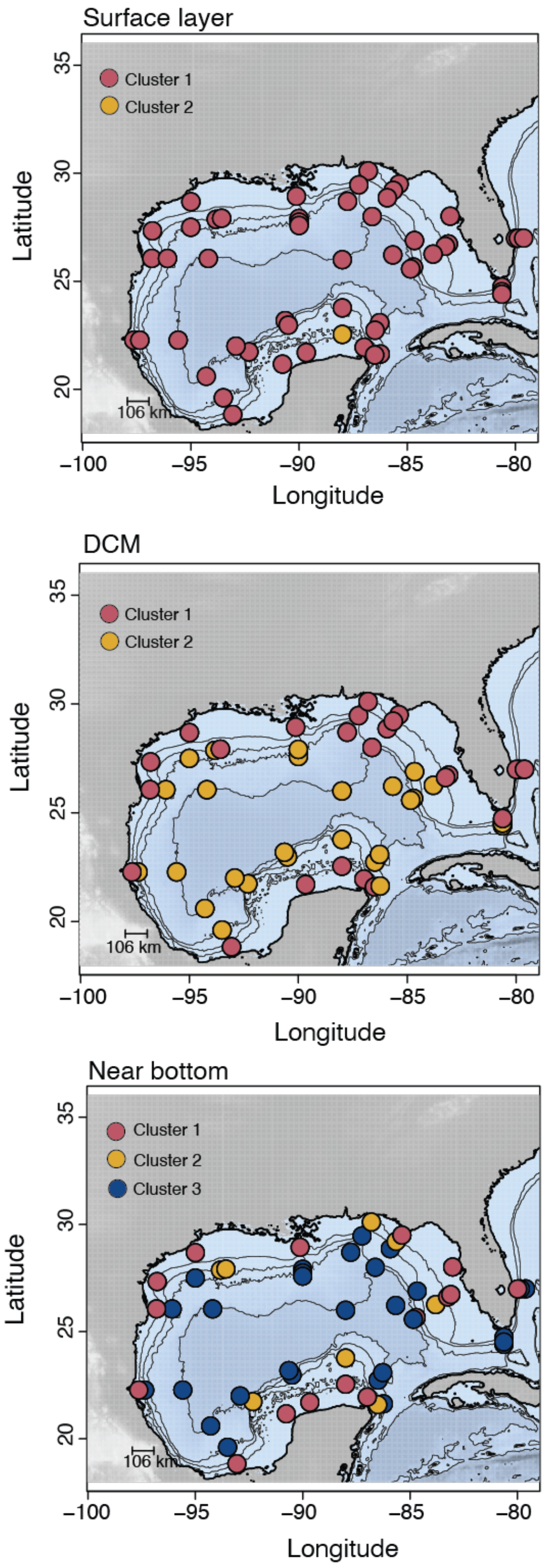

Surface layer
Cluster 1
Cluster 2
Latitude
35
30
25
20
106 km
−100
−95
−90
−85
−80
Longitude
DCM
Cluster 1
Cluster 2
Latitude
106 km
Longitude
Near bottom
Cluster 1
Cluster 2
Cluster 3
Latitude
106 km
Longitude

Figure S7: Distribution of 16S samples at the basin scale. Samples are colored as in Fig. 1 based on cluster assignments, showing lateral distribution patterns at the surface (top), deep chlorophyll maximum (DCM; middle), and near bottom (bottom). 16S sample clusters were in line with 18S (93% similar), with some differences in the photic zone (Cluster 1) and DCM (Cluster 2). Samples were collected in triplicate (shown as a single filled point), and environmental samples were collected from all sites. Isobaths are the same as in Fig. 1 and indicate 50, 200, 1000, and 3000 m depth.

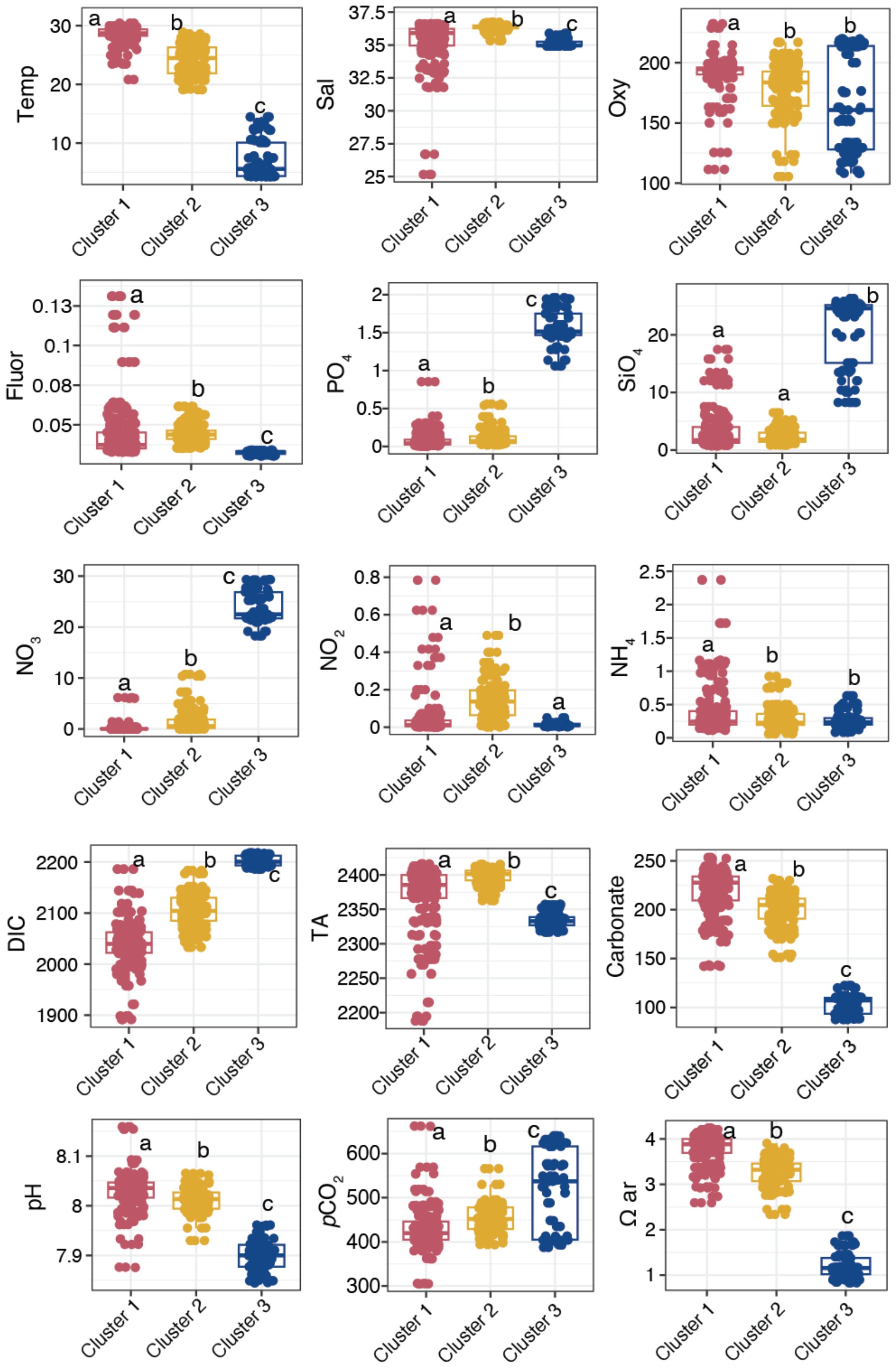

Temp
30
20
10
a
b
c
Sal
37.5
35
32.5
30
27.5
25
a
b
c
Oxy
200
150
100
a
b
b
Fluor
0.13
0.1
0.08
0.05
a
b
c
PO4
2
1.5
1
0.5
0
a
b
c
SiO4
20
10
0
a
a
b
NO3
30
20
10
0
a
b
c
NO2
0.8
0.6
0.4
0.2
0
a
b
a
NH4
2.5
2
1.5
1
0.5
0
a
b
b
DIC
2200
2100
2000
1900
a
b
c
TA
2400
2350
2300
2250
2200
a
b
c
Carbonate
250
200
150
100
a
b
c
pH
8.1
8
7.9
a
b
c
pCO2
600
500
400
300
a
b
c
Ω ar
4
3
2
1
a
b
c
Cluster 1
Cluster 2
Cluster 3

Figure S8: Differences in environmental parameters from Clusters 1–3, which reflect ecological depth zones in the GOM at the time of sampling. Cluster 1 = photic zone; Cluster 2 = deep chlorophyll maximum (DCM); Cluster 3 = aphotic zone. See Fig. 1 for the spatial distribution of DNA samples in each cluster. Boxplots show median values (horizontal line), with the boundaries representing the interquartile ranges (IQR 25–75%) and whiskers extending to 1.5 x the IQR. Data falling outside of the 1.5 x IQR are considered outliers in the plot. All data points are shown per cluster, including replicates. Letters denote statistical significance among means of different clusters ($P < 0.05$) and were estimated with Wilcoxon tests. Temp = temperature (°C); Oxy = oxygen (µmol kg^{-1}); Sal = salinity; Fluor = fluorescence; PO_4 = phosphate (µmol kg^{-1}); SiO_4 = silicate (µmol kg^{-1}); NO_3 = nitrate (µmol kg^{-1}); NO_2 = nitrite (µmol kg^{-1}); NH_4 = ammonium (µmol kg^{-1}); DIC = dissolved inorganic carbon (µmol kg^{-1}); TA = total alkalinity (µmol kg^{-1}); Carbonate (µmol kg^{-1}); $p\mathrm{CO}_2$ = partial pressure of CO_2 (µmol kg^{-1}); Ω ar = aragonite saturation state. These trends are presented for the 18S dataset, but clustering (Fig. S7) and patterns in environmental data were similar for 16S.

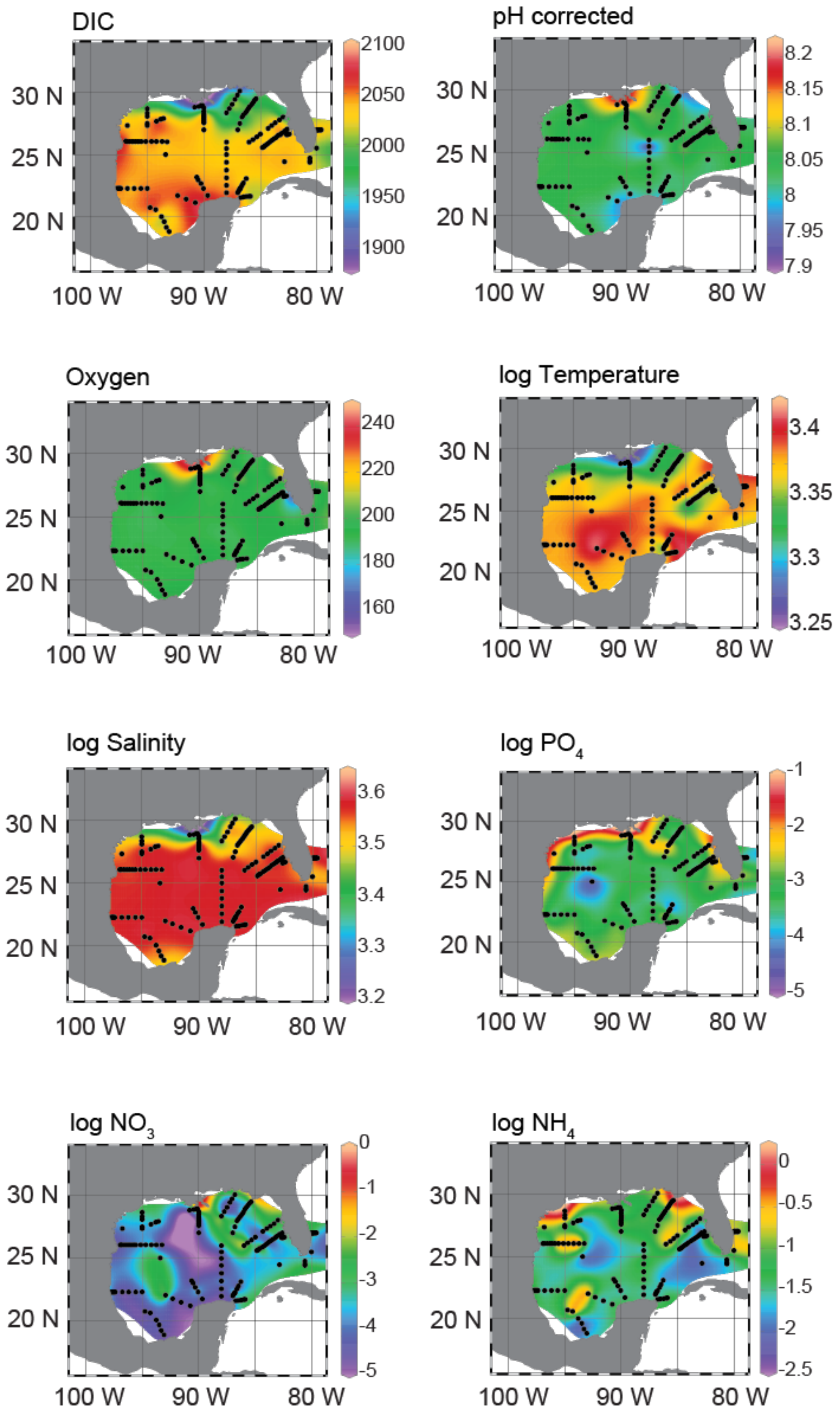

DIC
2100
2050
2000
1950
1900
30 N
25 N
20 N
100 W
90 W
80 W
pH corrected
8.2
8.15
8.1
8.05
8
7.95
7.9
Oxygen
240
220
200
180
160
log Temperature
3.4
3.35
3.3
3.25
log Salinity
3.6
3.5
3.4
3.3
3.2
log PO_4
-1
-2
-3
-4
-5
log NO_3
0
-1
-2
-3
-4
-5
log NH_4
0
-0.5
-1
-1.5
-2
-2.5

Figure S9: Spatial variability of environmental factors in the photic zone that were considered for microbial GAMs. Values for all 141 sites are shown, including 84 sites where only environmental variables (and no DNA samples) were measured. Temperature, salinity, PO_4, NO_3, and NH_4 were log transformed (with pseudo-count + 0.1) prior to GAMs to better normalize their distribution across the basin. Values have been interpolated using DIVA interpolation in Ocean Data View and linear color scales were applied for each variable (right of panel) to display units of measure from low (purple) to high (red) values. DIC = dissolved inorganic carbon and pH was corrected for *in situ* conditions. PO_4 = phosphate; NO_3 = nitrate; NH_4 = ammonium.

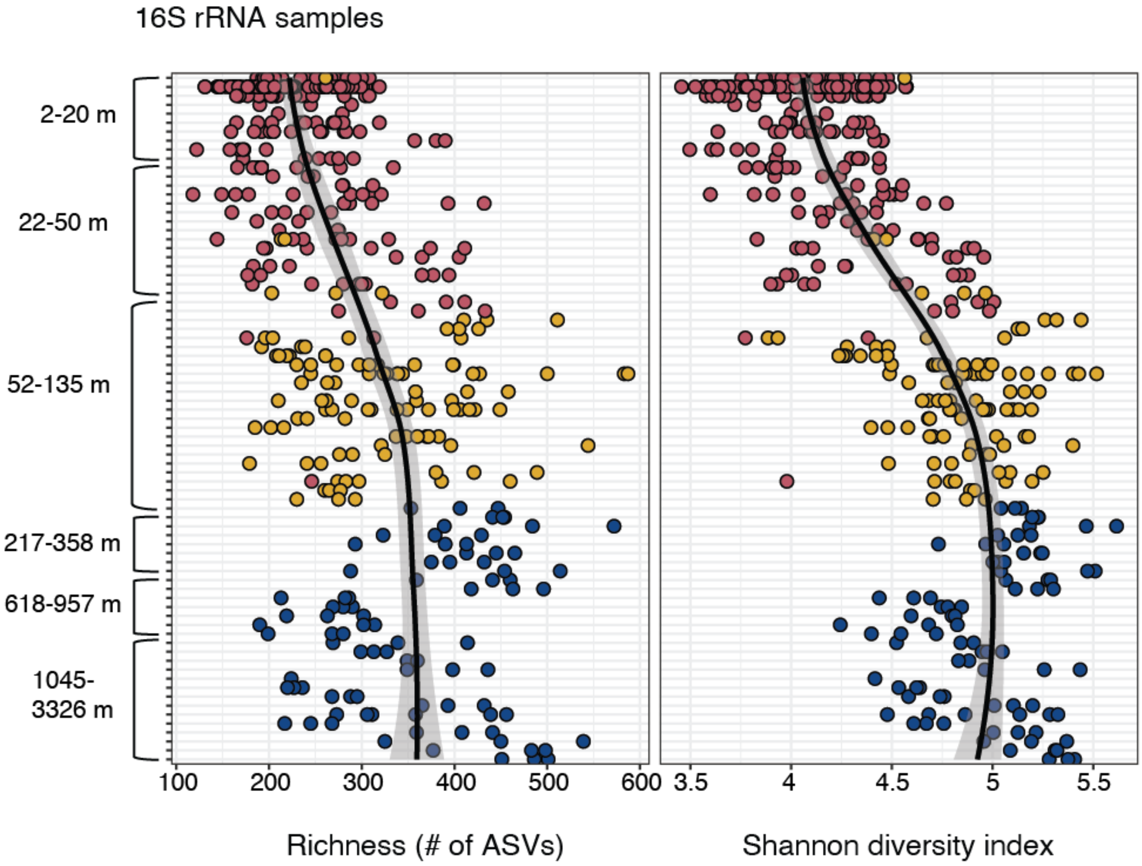

Figure S10: Species richness (# of ASVs) and Shannon diversity index for all 16S samples collected on the cruise with respect to absolute sampling depth. To show depth patterns, local regression (loess) curves were applied to the data and represent smoothed trends (black lines) with 95% confidence intervals. Samples are colored by cluster and absolute depth has been separated into multiple groups to better avoid overlap of discrete values on the y-axis. See Table S1 for sample metadata that includes depth and cluster information for each sample.

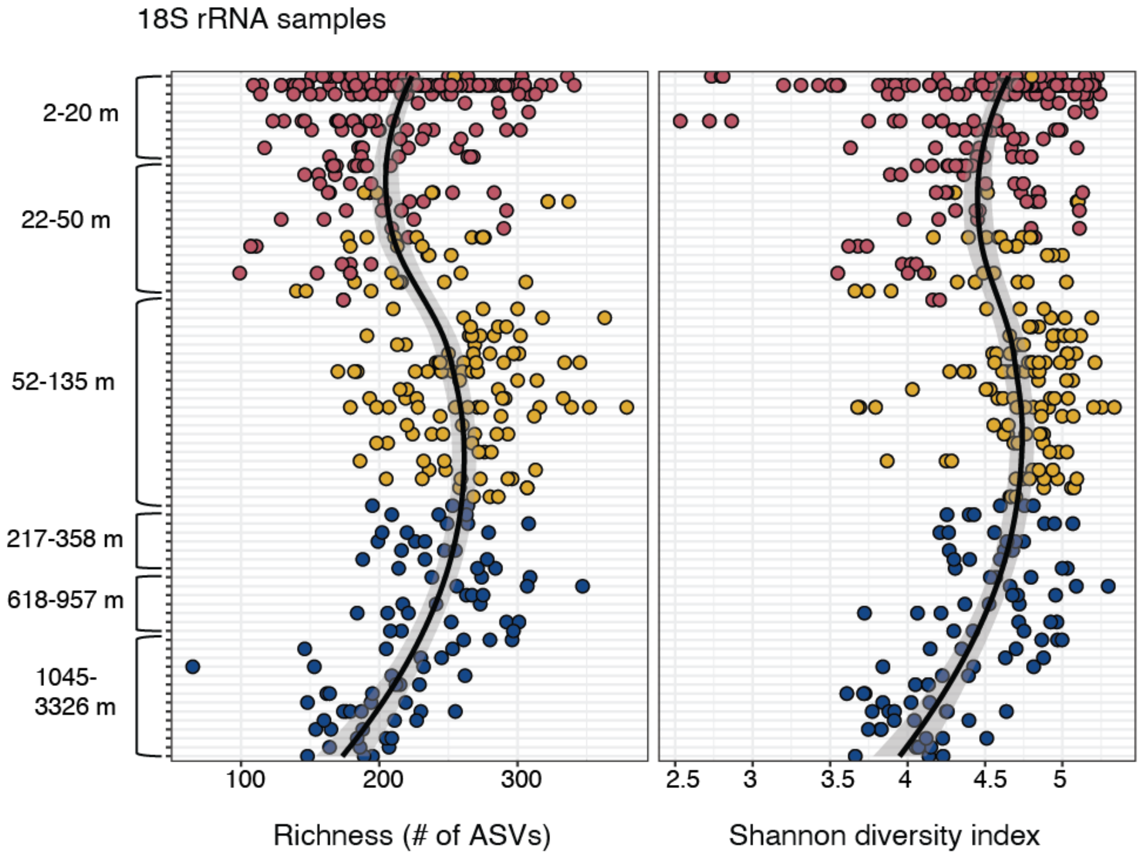

Figure S11: Same plot as in Fig. S10 but for 18S samples, highlighting trends in species richness and Shannon diversity with absolute sampling depth.

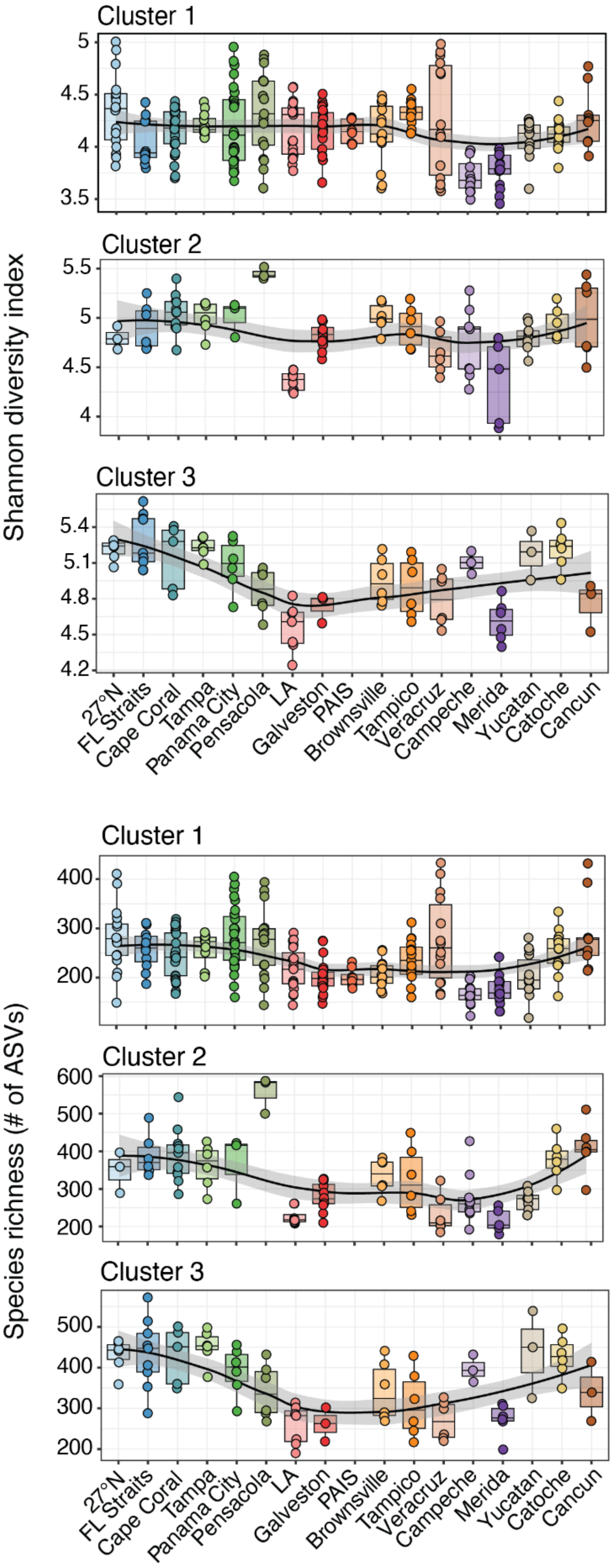
Cluster 1
Cluster 2
Cluster 3
Shannon diversity index
5
4.5
4
3.5
5.5
5
4.5
4
5.4
5.1
4.8
4.5
4.2
27°N
FL Straits
Cape Coral
Tampa
Panama City
Pensacola
LA
Galveston
PAIS
Brownsville
Tampico
Veracruz
Campeche
Merida
Yucatan
Catoche
Cancun
Cluster 1
Cluster 2
Cluster 3
Species richness (# of ASVs)
400
300
200
600
500
400
300
200
500
400
300
200
27°N
FL Straits
Cape Coral
Tampa
Panama City
Pensacola
LA
Galveston
PAIS
Brownsville
Tampico
Veracruz
Campeche
Merida
Yucatan
Catoche
Cancun

Figure S12: Shannon diversity index (top) and species richness (# of ASVs; bottom) for 16S samples with respect to transect and faceted for each of the three clusters (Clusters 1–3). Boxplots display median values (horizontal line), with boundaries representing the interquartile ranges (IQR 25–75%) and whiskers extending to 1.5 x the IQR. Data points outside of the 1.5 x IQR are considered outliers. All data points are shown for each transect. Local regression (loess) curves were applied to the data and represent smoothed trends (black lines) with 95% confidence intervals. Sampling lines are ordered counterclockwise in the Gulf according to the order of sampling; however, several lines (FL Straits and Cape Coral) were sampled last but grouped here with other Florida lines to more accurately visualize spatial patterns. LA = Louisiana and PAIS = Padre Island National Seashore.

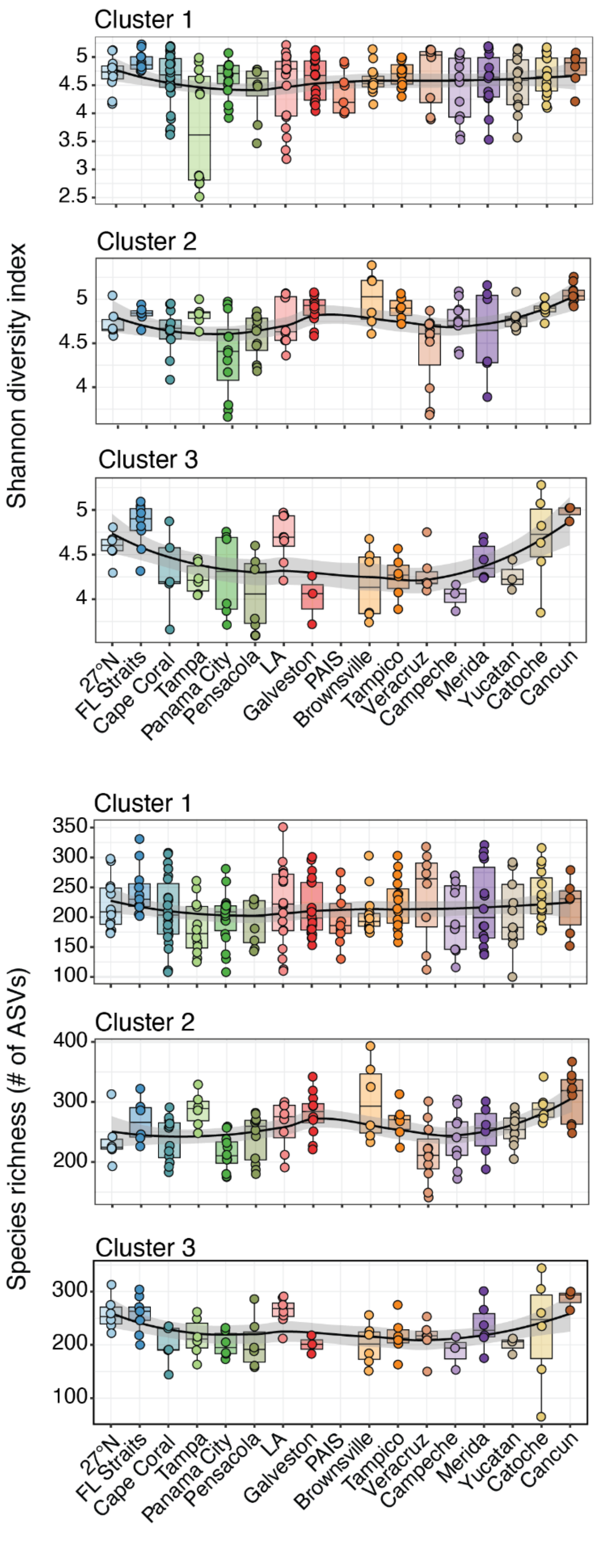
Cluster 1
Cluster 2
Cluster 3
Shannon diversity index
5
4.5
4
3.5
3
2.5
27°N
FL Straits
Cape Coral
Tampa
Panama City
Pensacola
LA
Galveston
PAIS
Brownsville
Tampico
Veracruz
Campeche
Merida
Yucatan
Catoche
Cancun
Species richness (# of ASVs)
350
300
250
200
150
100
400

Figure S13: Same plot as in Fig. S12 but for 18S samples, highlighting trends in Shannon diversity and species richness with respect to sampling transect and clusters.

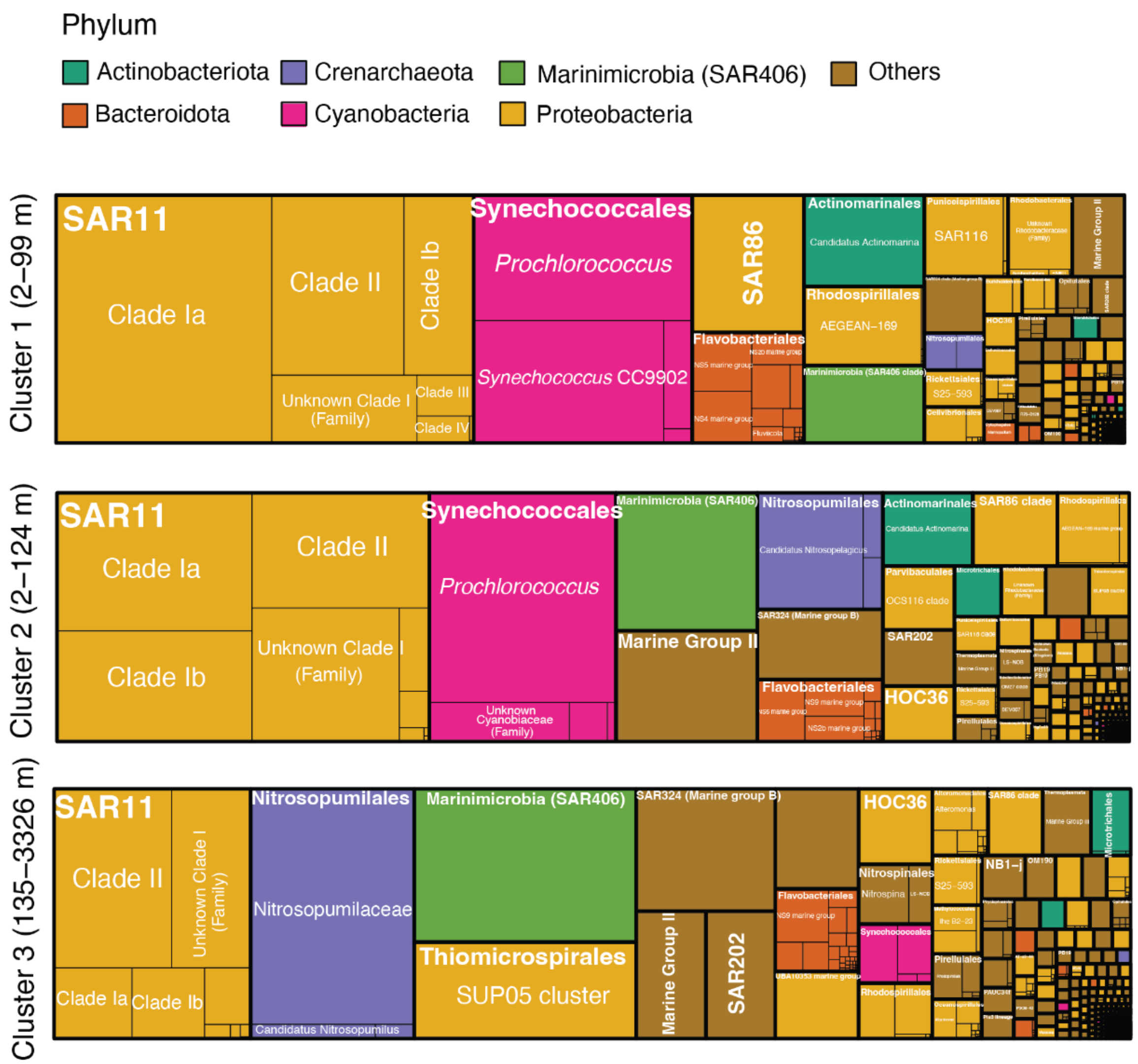

Figure S14: Taxonomy tree maps of 16S samples based on mean relative abundance (%) across all samples in Clusters 1–3. Taxonomic assignments were made via the SILVA database at the order level (larger boxes) and genus level within each order (smaller boxes). Boxes are colored by phylum and focused on the top six groups, with less abundant organisms at the phylum level grouped into an “others” category (in brown). Clusters largely reflected depth in the water column on the shelf vs. open ocean: Cluster 1 (photic zone; 2–99 m), Cluster 2 (DCM; 2–124 m), and Cluster 3 (aphotic zone; 135–3326 m).

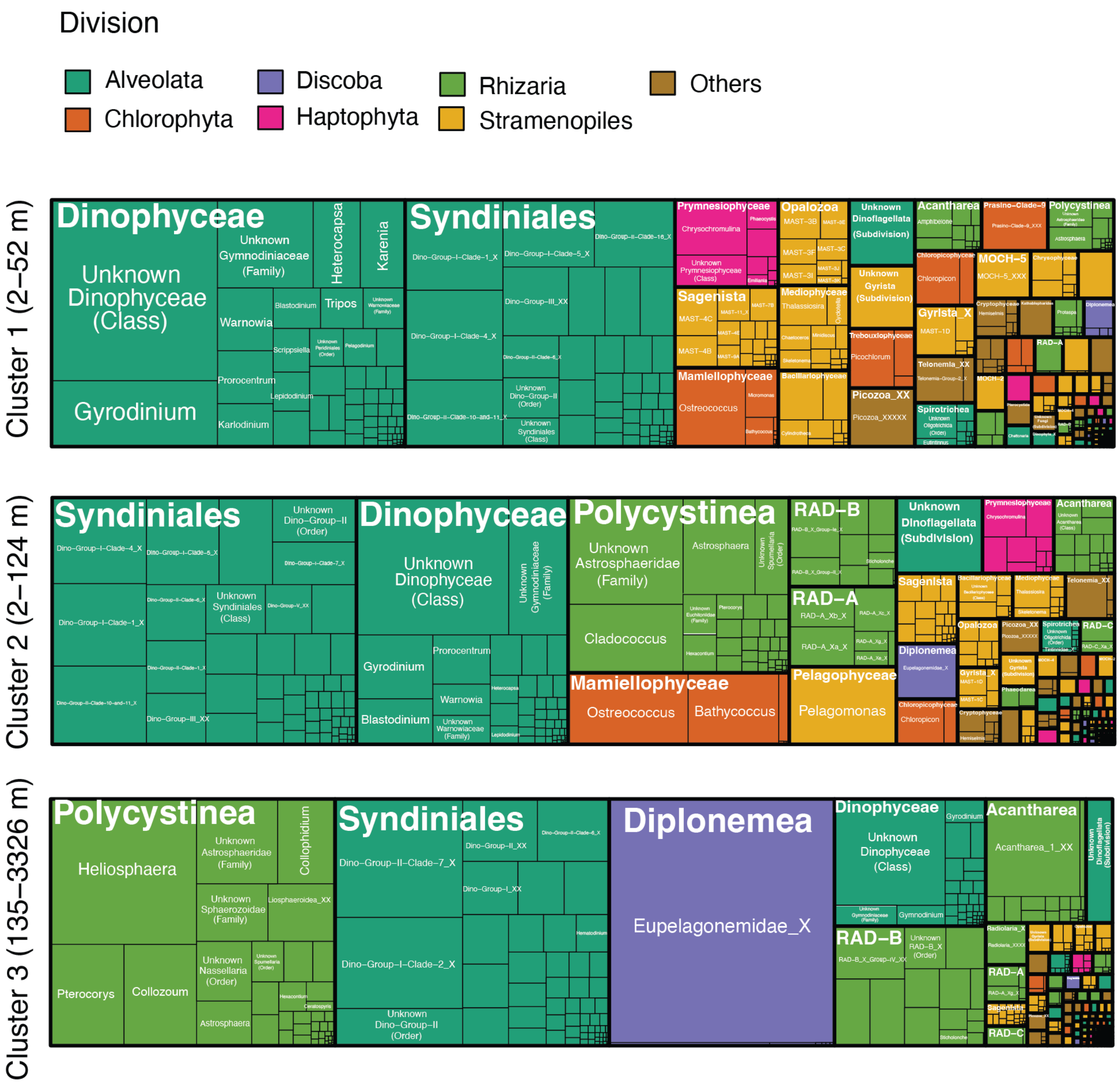

Figure S15: Taxonomy tree maps of 18S samples based on mean relative abundance (%) across all samples in Clusters 1–3. Taxonomic assignments were made via the PR2 database at the class level (larger boxes) and genus level within each class (smaller boxes). Boxes are colored by division and focused on the top six groups, with less abundant organisms at the division level grouped into an "others" category (in brown). Clusters were in line with those observed for 16S samples in Fig. S14.

Supplemental table legends:

Table S1: Environmental data measured at each site and depth that coincided with DNA sampling on GOMECC-4. Samples are distinguished by transect (region), station number, categorial depth (surface, DCM, and near bottom), and replicate (A–C). Near bottom is referred to as "deep" in the metadata and count table. Distance to shore indicates position of samples on the continental shelf (inshore; < 200 m) vs. in the open GOM (offshore; > 200 m). Volume filtered for discrete DNA samples is included, which was used to estimate DNA yield (ng liter^{-1}). The total alkalinity:dissolved inorganic carbon (TA:DIC) ratio was calculated and used for indicator analysis based on manual categories of low (< 1.16) vs. high TA:DIC (> 1.16). Cluster designations are also provided (Clusters 1–3) for 16S and 18S samples that were revealed through hierarchical clustering of community composition. "N/A" indicates an instance where a parameter was not measured.

Table S2: Functional assignments for all 18S and 16S (separate sheets) amplicon sequence variants (ASVs) that were identified in GOMECC-4 DNA samples. Shown for each ASV are the original sequence ID, sequential ASV number ("bASV" to discriminate Bacteria/Archaea in the 16S sheet), functional category, and taxonomic levels. Functional categories were manually assigned using a functional annotation repository that was previously applied to Tara Ocean 18S V9 samples: https://doi.org/10.5281/zenodo.3768950. See supplemental methods for additional details. Mixotrophic protists were categorized as constitutive mixotrophs (CM) that inherently have chloroplasts or endosymbiotic specialist non-constitutive mixotrophs (eSNCM) that harbor endosymbionts to support growth. Assignments were made before filtering out metazoans, and so, several metazoan groups are included here (copepods, gelatinous zooplankton, mollusks, and other metazoans) but were removed prior to downstream analysis. For prokaryotes, functional assignments included heterotrophic and autotrophic groups.

Table S3: Spearman rank correlations between environmental factors for 16S and 18S datasets in Clusters 1–3 (separate sheets). pH values were recalculated based on *in situ* conditions. TA = total alkalinity and DIC = dissolved inorganic carbon. Variables were selected for Cluster 1 models based on low collinearity (Spearman $r_s < |0.7|$) that resulted in a variance inflation factor (VIF) < 10. 16S and 18S datasets share the same metadata, but they were processed separately, resulting in slight differences in filtering (e.g., removal of certain groups, singletons, or rare taxa) and in the number and identity of samples retained in each dataset. This led to differences in the cluster assignments on a sample-by-sample basis, and therefore in the Spearman correlation values calculated per cluster. Assigned clusters were 93% similar between 16S and 18S samples.

Table S4: Akaike information criterion (AIC) values generated from different models of rarefied sequencing count data in the photic zone across major microbial groups. The following models were initially tested with non-collinear factors (see Fig. S2): generalized linear model (GLM) with Poisson distribution (GLM Poisson), GLM with negative binomial distribution (GLM NB),

generalized additive model (GAM) with negative binomial distribution (GAM NB), and GAM with Gaussian error distribution and either log transformed (GAM Gaussian log) or centered log-ratio (CLR) transformed reads (GAM Gaussian CLR). Except for Gaussian GAMs, raw (rarefied) reads were used with no other data transformation.

Table S5: Summary results of indicator analysis for 16S and 18S samples (separate sheets) based on high (> 1.16) or low (< 1.16) TA:DIC ratios in the photic zone. Indicator values are shown at the ASV level, along with taxonomic information for each 16S (phylum, genus, and species) and 18S ASV (division, genus, and species). Taxonomic levels were assigned via the PR2 and SILVA databases for 18S and 16S samples, respectively. Average relative abundance (%) of each ASV in the photic zone is also included, as well as the significance of the indicator value without and with correcting for multiple comparisons ($P < 0.05$; Benjamini-Hochberg correction). Prokaryotic ASVs are labeled as “bASV” to denote them from protists.
